# GLANCE-aided snapshotting for sustainable integration of synchronous spectrofluorimetry and micellar boosting for nanoscale assay of tolterodine binary mixtures in crucial matrices

**DOI:** 10.1038/s41598-025-27144-0

**Published:** 2025-12-05

**Authors:** Eman Yosrey, Heba Elmansi, Shereen Shalan, Jenny Jeehan Nasr

**Affiliations:** Department of Pharmaceutical Analytical Chemistry, Faculty of Pharmacy, Mansoura University, Mansoura, 35516 Egypt

**Keywords:** Synchronous spectrofluorimetry, Micellar enhancement, Tolterodine, Doxazosin, Terazosin, Sustainable chemistry, GLANCE tool, Chemistry, Environmental sciences

## Abstract

Tolterodine tartrate (TLD) has recently demonstrated remarkable synergism when combined with either of two α_1_-adrenergic antagonists, doxazosin mesilate (DXZ) or terazosin hydrochloride dihydrate (TRZ), for treating urinary tract issues. Herein, a sustainability-oriented approach is adopted for integrating constant wavelength synchronous fluorescence with micellar augmentation for ultra-trace tracking of TLD in combination with DXZ (mixture I) or TRZ (mixture II). The designed protocol relies chiefly on exploiting sodium dodecyl sulfate as a biodegradable fluorescence enhancer and water as an eco-friendly diluting solvent, maintaining a fixed wavelength difference (Δλ = 20 nm) to record the drugs’ signals. Under optimal experimental conditions, favorable linearity at the nano level was achieved over concentration ranges of 20.0–200.0, 5.0–50.0, and 5.0–50.0 ng mL⁻^1^, with remarkable LOD (1.25, 0.90, and 0.73 ng mL⁻^1^) and LOQ (3.78, 2.73, and 2.20 ng mL⁻^1^) levels for TLD, DXZ, and TRZ, respectively. The methodology effectively evaluated the cited drugs *per se*, in their laboratory-prepared mixtures, and in dosage form, without impediments from one another or the tablet excipients. The outstanding ultra-sensitivity of the developed strategy permitted concurrent minute analysis of the targeted drugs in human plasma, with acceptable standard deviation (SD) values ≤ 2.40. Additionally, exceptional analytical performance has been demonstrated for the nominated medications in two environmental samples with SD values ranging from 0.59 to 1.57. The proposed work prioritizes sustainability principles by excluding harsh chemicals and convoluted procedures, constituting a cornerstone of the design. These principles were checked using: MoGAPI, AGREE, CACI, RGBfast, RAPI, and BAGI. Interestingly, the cutting-edge GLANCE tool was employed to display a quick snapshot of the framework aspects in a well-organized template, without digging into overwhelming datasets. The outcomes demonstrated the excellence of the designed methodology over reported approaches, offering proficient sensitivity, sustainability, facility, and practicability across diverse crucial matrices. These findings provide compelling evidence that the proposed work serves as an efficient, eco-friendly quantification tool for use in quality control, biological, and environmental sectors, fostering a more eco-conscious and sustainable scientific community.

**Supplementary Information:**

The online version contains supplementary material available at 10.1038/s41598-025-27144-0.

## Introduction

One of the most intricate and prevalent problems affecting men’s health is lower urinary tract symptoms, including frequent urination, nocturia, and a sensation of incomplete emptying. Clinical publications have reported the occurrence of these complications in men who use ureteral stents or suffer from benign prostatic hyperplasia (BPH)^1,2^. Recent studies suggest that combining muscarinic receptor antagonists, such as tolterodine tartrate, with α_1_-adrenergic receptor antagonists, such as doxazosin mesylate, can help alleviate these symptoms in BPH patients. Similarly, the combination of tolterodine tartrate with terazosin hydrochloride dihydrate has shown benefits for patients using ureteral stents. These investigations have also demonstrated the effectiveness of such combinations compared to their individual use^1,2^. Therefore, it is crucial to develop a reliable and precise analytical method for measuring these drugs in biological fluids, serving as a follow-up tool to monitor patient adherence to medication.

**Fig. 1:**
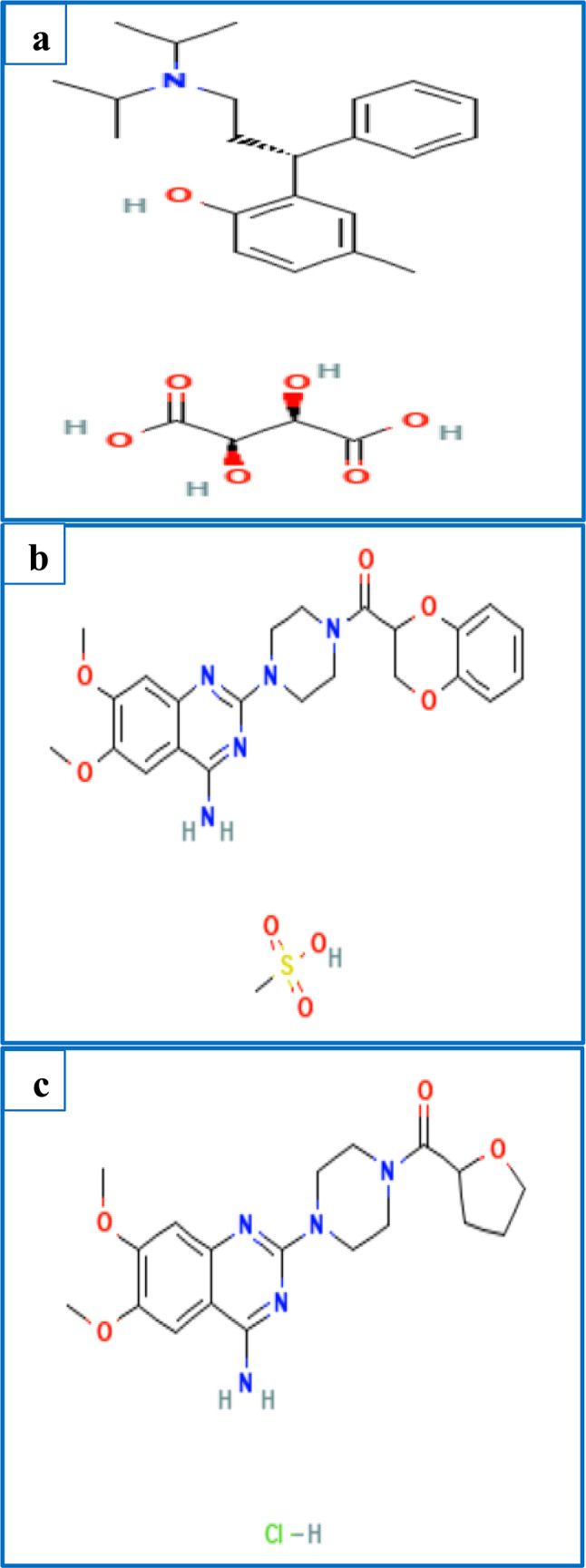
Chemical Structure of (**a**) TLD, (**b**) DXZ, and (**c**) TRZ.

Tolterodine tartrate (TLD, Fig. 1a) is an antimuscarinic medication that is primarily prescribed for treating overactive bladder syndrome, a troublesome chronic disorder signified by urinary urgency and increased frequency. TLD reduces bladder contractions by inhibiting the tone of smooth muscles. Its chemical name is ( +)-(R)-2-p-cresol tartrate^3^. Doxazosin mesilate (DXZ, Fig. 1b) functions as a selective α_1_-adrenergic receptor antagonist and is prescribed for BPH patients to reduce urethral resistance and improve urinary flow. It is chemically designated as 1-(4-Amino-6,7-dimethoxyquinazolin-2-yl)-4-[(2RS)-2,3-dihydro-1,4-benzodioxin-2-ylcarbonyl] piperazine methanesulfonate^4^. Another selective α_1_-adrenergic receptor antagonist, terazosin hydrochloride dihydrate (TRZ, Fig. 1c), is used for patients with ureteral stents to alleviate bladder outlet resistance, urine reflux, and flank pain. It is chemically named 1-(4-Amino-6,7-dimethoxyquinazolin-2-yl)-4-[[(2RS)-tetrahydrofuran-2-yl] carbonyl] piperazine hydrochloride dihydrate^4^.

In the realm of analytical methods, various quantification procedures have been published for monitoring TLD, including spectrophotometry^5–7^, spectrofluorimetry^8,9^, thin-layer chromatography^10,11^, HPLC^12,13^, capillary electrophoresis^14,15^, and electrochemical methods^16–18^. Regarding DXZ, several analytical approaches have also been documented, including spectrophotometry^19,20^, spectrofluorimetry^21–23^, thin-layer chromatography^24,25^, HPLC^26,27^, capillary electrophoresis^28^, and electrochemical methods^20,29^. For TRZ, the literature reveals a variety of analytical procedures, including spectrophotometry^30,31^, spectrofluorimetry^22,23^, thin-layer chromatography^24,25^, HPLC^26,32^, and voltammetry^33^.

However, chromatographic methods^12,13,26,27,32^ rely heavily on organic solvents, which are environmentally hazardous, pose safety risks to analysts, increase the analysis costs and generate substantial waste. Additionally, these methods typically require multi-step preconditioning, which reduces analysis productivity. Meanwhile, electrochemical^16,17,20,29,33^ and electrophoretic^14,15,28^ procedures are hampered by their complexity, requiring advanced expertise, along with low throughput. The reported spectrofluorimetric methodologies are also constrained by their lengthy procedures, which can take up to 45 minutes^22^, and their reliance on environmentally harmful organic reagents and solvents^8,21,22^ (such as dansyl chloride, orthophthalaldehyde, fluorescamine, and dichloromethane).

To date, no studies have investigated the synchronous spectrofluorimetric properties of TLD, DXZ, or TRZ, with only a single publication addressing their intrinsic fluorescence characteristics^9,23^. Therefore, the proposed approach aims to fill this gap and overcome the limitations of the previous works by proposing facile, sensitive, and eco-friendly synchronous spectrofluorimetric procedures that permit the simultaneous recording of TLD in the presence of either DXZ or TRZ in crucial matrices. Practically, spectrofluorimetry stands out among analytical techniques due to its low cost and high sensitivity for detecting fluorescent analytes. It enables detection in the nano-gram range, offering a promising strategy for quantifying pharmaceuticals in biological samples. Furthermore, it is noted for its rapid analysis and user- and environmentally friendly nature, requiring minimal or no harmful organic solvents and consuming less than 0.1 kV per sample. Additionally, the method is non-destructive, which qualifies it to be a prime tool for analyzing intricate and delicate samples. Nonetheless, selectivity issues may arise when analyzing multi-component mixtures due to overlapping broadband spectra. This challenge can be mitigated by employing constant-wavelength synchronous fluorescence spectroscopy (CWSS), in which excitation and emission monochromators are scanned simultaneously while maintaining a constant-wavelength interval (∆λ) between excitation and emission wavelengths, resulting in sharper and narrower spectral peaks. Over time, the adoption of CWSS through discriminating matrices has garnered sustained interest among analytical chemists as a fingerprinting tool for selectively resolving multi-analyte mixtures. Integrating CWSS with derivatized signals further reduces light scattering, generates simpler spectra and enhances resolution, thus improving selectivity. These virtues make this integration an ideal protocol for the sensitive and selective concurrent tracking of multi-component mixtures without requiring prior separation steps^34–36^.

Global attention has increasingly focused on monitoring pharmaceuticals in aquatic systems, as it has been acknowledged that conventional water treatment processes are not specifically designed to eliminate these contaminants. Recent studies highlighted that pharmaceuticals are often released into the environment, primarily due to manufacturing processes and the disposal of unused or expired medications. Several environmental studies have confirmed the detrimental impact of pharmaceutical residues, even at ng L^−1^ concentration levels, on aquatic ecosystems^37,38^. Consequently, there is an urgent need to establish sensitive and reliable analytical strategies for detecting pharmaceuticals in water systems. . With minimal sample consumption, rapid scanning capability, and requiring only simple filtration as a pretreatment step, the proposed work is a pioneering effort toward point-of-care testing in water analysis. In this context, the developed methodology assesses two environmental water samples, marking a critical milestone in supporting analytical environmental studies^39–41^.

In light of these challenges, introducing validated and specific analytical procedures for concurrent analysis of trace levels of the nominated targets across diverse matrices is both beneficial and mandatory. The primary goal of this study is to establish a streamlined framework capable of detecting ultra-trace quantities of TLD, DXZ, and TRZ, either as standalone medications in their raw materials, dosage forms, and environmental water samples, or as co-administered drugs in binary mixtures in biological fluids. This framework aims to consolidate the merits of deconvoluted CWSS with micellar-boosted fluorescence, while adhering to green analytical chemistry (GAC), sustainability and functionality principles. This was accomplished through reliance on direct scanning using sodium dodecyl sulfate (SDS) enhancer and water as a diluting solvent, which are viable for analytical practices in resource-limited laboratories. This helped in facilitating workflows, conserving resources, and decreasing the need for extra materials and energy. The designed framework was evaluated using a plethora of recently introduced assessment tools as an essential step to provide a comprehensive representation of its greenness, whiteness and blueness profiles.

## Experimental

### Materials and reagents

All chemicals and reagents were of analytical grade:

Tolterodine tartrate (purity 99.55%) was obtained from El-Kahira (Cairo, Egypt). Doxazosin mesilate (purity 99.82%) and terazosin hydrochloride dihydrate (purity 99.92%) were obtained from Pharaonia Pharmaceuticals (Alexandria, Egypt). Britton Robinson buffer (BRB) ingredients of acetic acid, boric acid, phosphoric acid and sodium hydroxide (El-Nasr Pharmaceutical Chemicals Co., Egypt). SDS, cetrimide, carboxy methyl cellulose (CMC), and Tween 80 (El-Nasr Pharmaceutical Chemicals Co, Egypt). Brij 35 and β-cyclodextrin (β-CD) (Sigma-Aldrich, Germany). HPLC-grade solvents, including methanol, ethanol, and acetonitrile (Fisher Scientific, UK).

For pharmaceutical formulation included Incont L. A^®^ tablets (batch No. 01241535), each contains 4 mg TLD/tablet (Adwia Pharmaceuticals, Egypt), Dosin^®^ tablets (batch No. 2304490), each contains 1 mg DXZ/tablet (Egyptian International Pharmaceuticals Industries Company (EIPICO), Egypt), and Itrin^®^ tablets (batch No. 41058), each contains 2 mg TRZ /tablet (Kahira Pharmaceuticals & Chemicals. Industries. Co, Egypt). The samples of human plasma were obtained from Mansoura University Hospital and kept refrigerated. The samples of Nile River water were obtained 2 m from the shore in a (1 L) clean amber glass bottle pre-rinsed with distilled water. The bottle was filled, sealed, and stored in a refrigerator until analysis. A laboratory tap water sample was collected after permitting it to run for 10 min.

### Instrumentation

The spectrofluorimetric studies were implemented employing a Cary Eclipse fluorescence spectrophotometer (Agilent Technologies, USA), equipped with a xenon flash lamp. A Centrifuge (2-16P, Sigma, Germany), vortex mixer (VM-300 P, GEMMY, Taiwan), and pH meter (NV P-901, Consort, Belgium) were used.

### Preparation of standard solutions

Standard stock solutions of TLD, DXZ, and TRZ (100.0 µg mL^−1^) were prepared by combining 10 mg of each raw material with methanol in 100 mL volumetric flasks. Appropriate dilutions were made to obtain standard working solutions of 500.0 ng mL^−1^ for both DXZ and TRZ and 2.0 µg mL^−1^ for TLD, using methanol as a diluent. All the prepared solutions were stored in the refrigerator at 4 °C.

### General procedures

#### Calibration curves

Suitable quantities of standard working solutions were added to three sets of 10 mL volumetric flasks to achieve concentration ranges of 20.0–200.0 ng mL^−1^ for TLD, 5.0–50.0 ng mL^−1^ for both DXZ , and TRZ . Subsequently, 1.6 mL of a 2.0 w/v% SDS solution was added to each flask and mixed thoroughly. Distilled water was then added to each solution up to the final mark. Synchronous signals were recorded at a ∆λ of 20 nm for all the studied drugs. The first derivative amplitudes of synchronous fluorescence intensities (DSFI) of the prepared solutions were measured at 292 nm for TLD, and at 355 nm for DXZ and TRZ, following the exclusion of the blank reading. Calibration curves were created by plotting the DSFI against the drug concentration in ng mL^−1^, and the regression equations were derived.

#### Laboratory-prepared mixtures

Binary laboratory-synthesized mixtures of TLD with DXZ (mixture I) and TLD with TRZ (mixture II) were prepared at varying ratios. The appropriate aliquots of standard working solutions were transferred to two sets of 10.0 mL volumetric flasks and then underwent analysis as per the previously mentioned steps in Sect. “Calibration curves”. The percentage recoveries and standard deviation (SD) values were calculated following the corresponding regression equation for each drug in both studied mixtures.

#### Pharmaceutical dosage forms

Ten tablets of each drug were accurately weighed and crushed to prepare powders for testing. The precise weights of the powders, equivalent to 5 mg for each drug, were individually transferred to 100 mL volumetric flasks containing approximately 50 mL of methanol. The flasks were sonicated for 30 min to achieve maximum solubilization of the targeted drugs, after which the flasks were topped up to the volume with the same solvent. Test solutions were obtained by filtering the resulting solutions and discarding the first portions. Ultimately, the assaying steps were performed according to the procedures outlined in Sect. “Calibration curves”. Using the corresponding regression formulas for each drug, the percentage recoveries for the nominal amounts and SD were calculated.

#### Content uniformity testing

Ten tablets of each drug were separately investigated for content uniformity testing following USP specifications^42^. The steps detailed in Sect. “Pharmaceutical dosage forms” were implemented to check the content of each tablet. Suitable volumes from the filtered stock solution equivalent to 100.0 ng mL^−1^ for TLD and 40.0 ng mL^−1^ for DXZ and TRZ were individually transferred to 10 mL volumetric flasks. The analytical study was performed according to the steps listed in Sect. “Calibration curves”. The percentage recovery for each tested tablet was computed according to the corresponding regression equations, and the acceptance value was then calculated for each analyte.

#### Biological fluids analysis

One mL of human plasma was transferred to two sets of 15 mL centrifugation tubes and spiked with appropriate aliquots of TLD with DXZ standard solutions to achieve final concentration ranges of (40.0–140.0) and (10.0–40.0) ng mL⁻^1^ for TLD and DXZ, respectively, for the analysis of mixture I. Regarding the analysis of mixture II, the plasma samples were spiked with appropriate aliquots of TLD with TRZ standard solutions to achieve final concentration ranges of (60.0–200.0) and (10.0–50.0) ng mL⁻^1^ for TLD and TRZ, respectively. The two sets were subsequently filled to 5.0 mL with acetonitrile to precipitate the proteins^34^. The tubes were vortexed for two minutes, followed by centrifugation at 4800 rpm for half an hour to separate the precipitated plasma proteins. The supernatant was passed through a 0.45 µm syringe filter, and one mL was transferred from each tube into a 10.0 mL volumetric flask. The procedures were carried out as previously stated under Sect. “Calibration curves”. Blank was performed in the same manner except for the drug addition. The DSFI of the signals was computed and graphed against the drug concentration in ng mL^−1^ to construct the matrix-matched calibration graph and calculate the percentage recoveries with SD values.

#### Environmental water analysis

Two water samples (river water and laboratory tap water) were kept aside to enable the suspended particles to settle, followed by filtration using filter paper. One mL of the filtered water sample was added to 10 mL volumetric flasks, then spiked with incremental aliquots of each targeted drug, to achieve final concentration ranges of (40.0–120.0) for TLD in both water samples, and final concentration ranges of (10.0–40.0) for DXZ and TRZ in both water samples. The procedure was implemented as aforementioned in Sect. “Calibration curves”. Matrix-matched calibrations were implemented for each analyte in both matrices, and DSFI was plotted *versus* the concentration of each drug in ng mL^−1^ to establish the regression equation and compute the percentage recoveries.

## Results and discussion

Initial examination of the native fluorescence spectra of TLD, DXZ, and TRZ revealed distinct emission peaks at 310, 400, and 401 nm, with λ_ex_ values of 225, 247, and 249 nm, respectively (Fig. S1). However, spectral overlap between the excitation spectra of DXZ and TRZ with both the excitation and emission spectra of TLD caused a reduction in the fluorescence emission intensity of TLD when recorded simultaneously with either DXZ or TRZ, due to the inner filter effect^43^. This effect occurs when the absorption spectrum of the quencher (DXZ or TRZ) overlaps with the excitation and/or emission spectra of the fluorophore (TLD). Such overlap complicates the accurate tracking of individual analyte concentrations in the investigated mixtures. To overcome this issue, a different scanning framework, CWSS, was adopted to allow the simultaneous analysis of the targeted analytes without mutual interference. Although CWSS reduced the spectral overlap, slight interference remained between the targeted compounds in both mixtures (Figs. S2 and S3). To resolve this, the first derivative amplitude of the CWSS for the studied drugs was computed, yielding deconvoluted spectral peaks with zero-crossing points that facilitate the selective identification of the introduced targets without interference.

### Conditions study and tuning

To refine the spectroscopic strategy concerning the optimal resolution and sensitivity of the two binary mixtures, the One-Variable-At-a-Time (OVAT) approach was followed, in which one variable is changed while keeping other factors constant. Various trials were executed to define the most suitable conditions, including ∆λ, diluting solvent, micellar systems, and pH impact.

#### Delta lambda

The selection of a suitable ∆λ is a pivotal factor in the CWSS approach, as it influences peak resolution, signal sensitivity, spectral shape, and the noise level. Several ∆λ values were tested, ranging from 20 to 120 nm. The results revealed that a ∆λ of 20 nm was suitable for both studied binary mixtures, providing optimal signal intensity, peak shape, and resolution spectra for these mixtures.

**Fig. 2:**
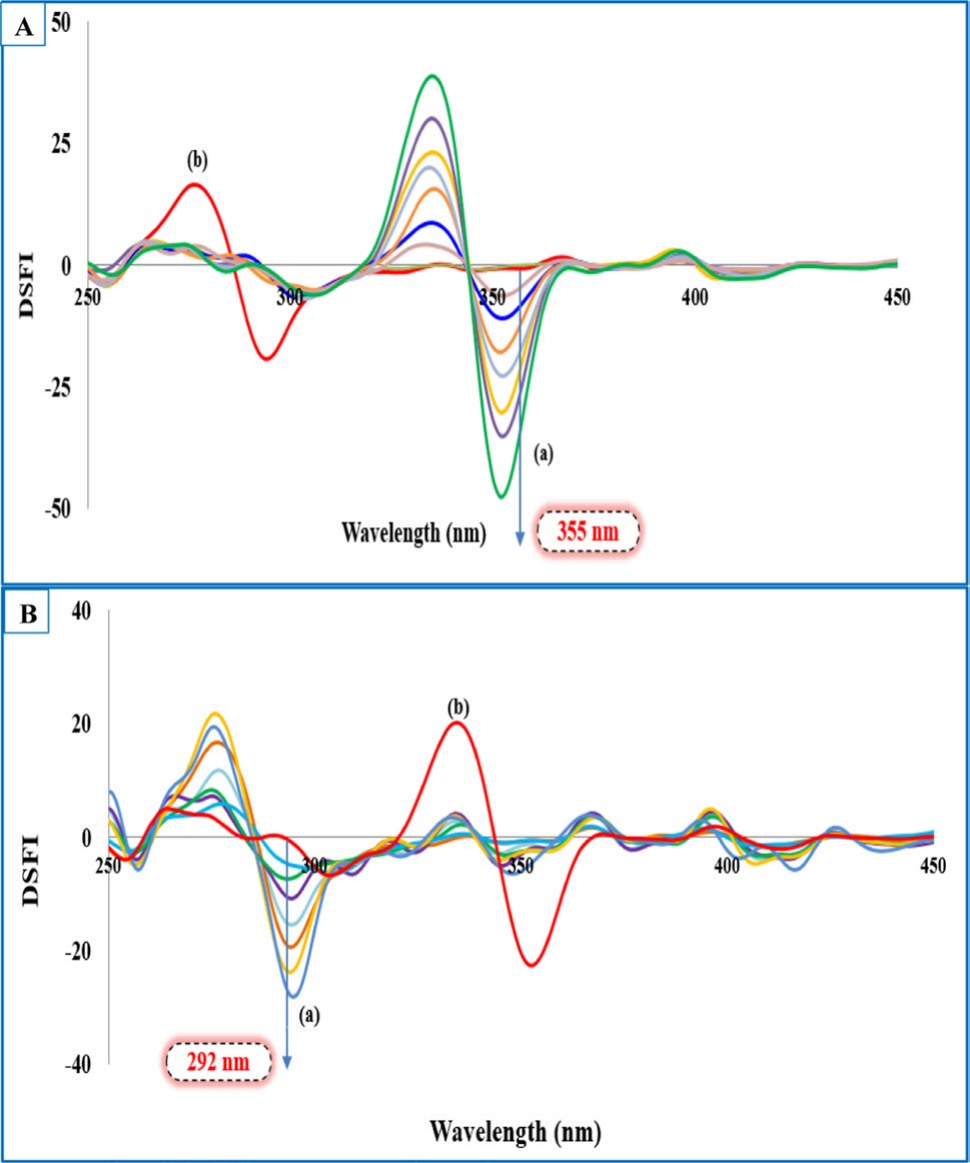
(**A**) First derivative synchronous fluorescence spectra at Δλ = 20 nm of: (**a**) DXZ (5.0, 10.0, 15.0, 20.0, 25.0, 30.0, 40.0, 50.0) ng mL⁻^1^. (**b**) TLD 120.0 ng mL⁻^1^. (**B**) First derivative synchronous fluorescence spectra at Δλ = 20 nm of: (**a**) TLD (20.0, 40.0, 60.0,100.0, 120.0, 160.0, 200.0) ng mL⁻^1^. (**b**) DXZ 20.0 ng mL⁻^1^.

**Fig. 3:**
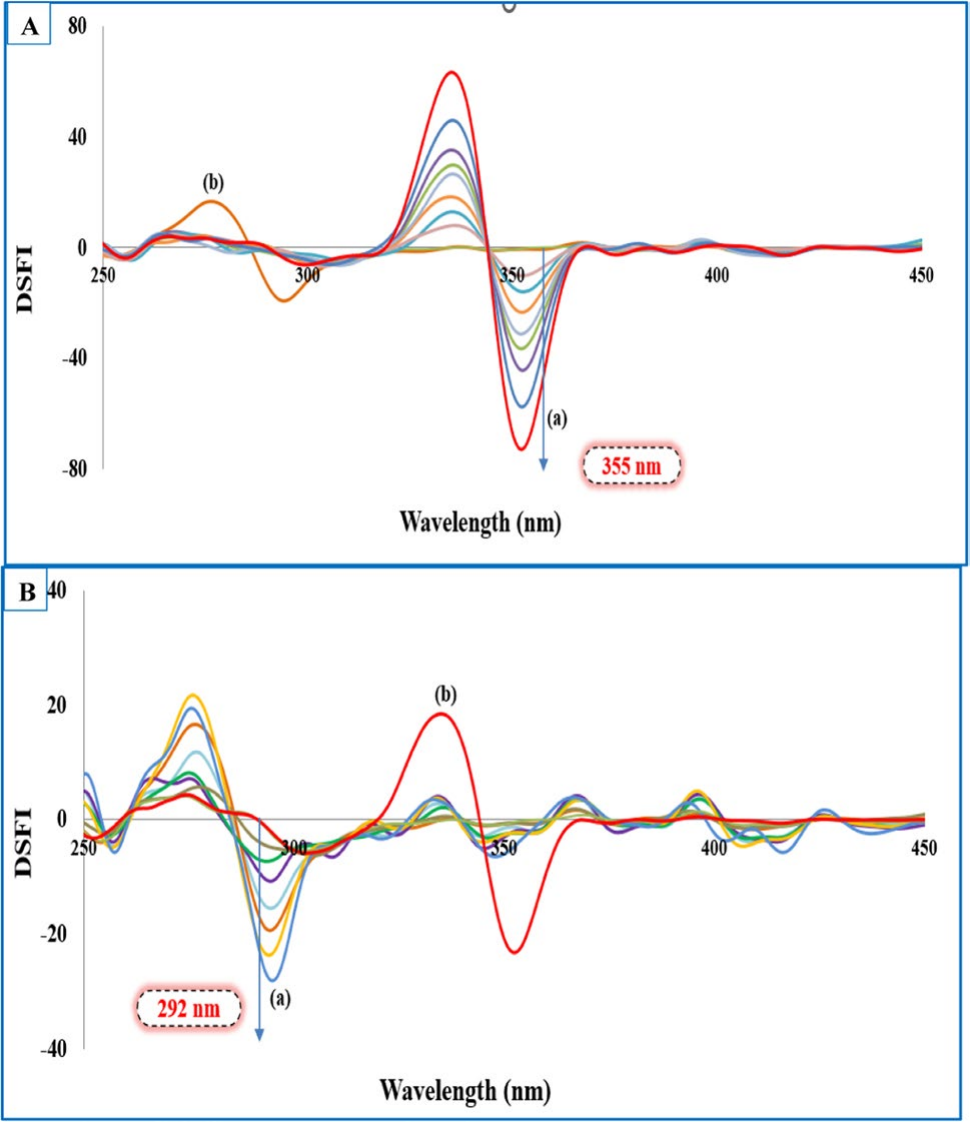
(**A**) First derivative synchronous fluorescence spectra at Δλ = 20 nm of: (**a**) TRZ (5.0, 10.0, 15.0, 20.0, 25.0, 30.0, 40.0, 50.0) ng mL⁻^1^. (**b**) TLD 120.0 ng mL⁻^1^. (**B**) First derivative synchronous fluorescence spectra at Δλ = 20 nm of: (**a**) TLD (20.0, 40.0, 60.0, 100.0, 120.0, 160.0, 200.0) ng mL⁻^1^. (**b**) TRZ 15.0 ng mL⁻^1^.

Nonetheless, the issue of spectral overlap could not be fully resolved as presented in Figs. S2 and S3. This was quantified using signal-to-background (S/B) ratios, which indicated partial interference: for the TLD–DXZ mixture, S/B was 0.55 (DXZ relative to TLD) and 1.01 (TLD relative to DXZ); for the TLD–TRZ mixture, S/B was 0.79 (TRZ relative to TLD) and 0.88 (TLD relative to TRZ). Combining the first derivative technique with the obtained synchronous fluorescence spectra allows attaining a zero-crossing point for each target in the two binary mixtures without mutual interference. The spectra produced in Figs. 2 and 3 revealed resolving the challenged spectral overlap, which could be selected as an ideal tool for selective monitoring of the targeted drugs.

#### Diluting solvent and organized media

**Fig. 4:**
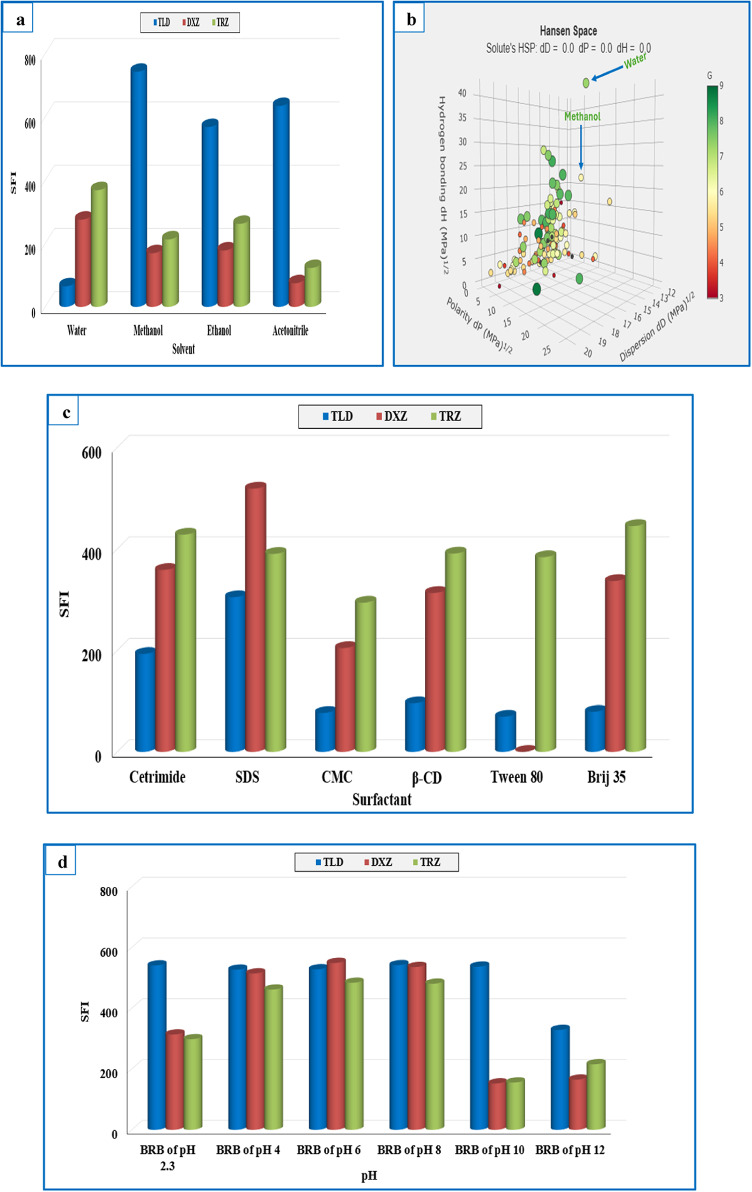
(**A**) Effects of different solvents on the SFI of 400.0 ng mL⁻^1^ of TLD, 40.0 ng mL⁻^1^ of DXZ, and 20.0 ng mL⁻^1^ of TRZ. (**B**) GSST chart for appraising water and methanol. (**C**) Effects of different micellar systems on the SFI of 400.0 ng mL⁻^1^ of TLD, 40.0 ng mL⁻^1^ of DXZ, and 20.0 ng mL⁻^1^ of TRZ. (**D**) Effects of different pH on the SFI of 400.0 ng mL⁻^1^ of TLD, 40.0 ng mL⁻^1^ of DXZ, and 20.0 ng mL⁻^1^ of TRZ.

The influence of solvent polarity and the surrounding environment on the shape, sensitivity, and resolution was evaluated using highly polar solvents (water, methanol, and ethanol) and moderately polar solvents (acetonitrile). This focus was attributed to polar solvents’ ability to stabilize the fluorophore’s excited state through solvation, which increases the fluorescence intensity. Non-polar solvents (like toluene and hexane) are known to promote non-radiative processes and decrease fluorescence intensity; hence, they were excluded. The results shown in Fig. 4a revealed that methanol produced the highest synchronous fluorescence intensity (SFI) for TLD peaks (with greater than 900% fluorescent enhancement compared with water), while water was the best for DXZ and TRZ (with 162% and 173% fluorescent enhancement compared with methanol). In this sequence, we rendered the Green Solvent Selection Tool (GSST) to evaluate the greenness properties for water and methanol. GSST is an innovative solvent evaluation tool introduced by Christian Larsen for nominating environmentally sustainable solvents based on GlaxoSmithKline’s solvent sustainability recommendations^44^. The evaluation is performed through free online software and represented as a numerical score (G) following the formula G= $\sqrt[4]{H\times S\times E\times W}$ where G stands for the fourth root of the outcome of the four primary ecological criteria, including Environment (E), Waste Disposal (W), Safety (S), and Health (H). The score ranges from 1 to 10, where highly sustainable solvents are ranked high, and lower grades indicate unsustainable ones. Besides the scoring index, each solvent is depicted visually by a sphere coded with a traffic-light color. A larger and greener sphere with a G score ≥ 7 points outlines the eco-friendliness features of the solvent and vice versa. The benefit of the GSST lies in its free availability and ease of use, which qualify the tool as a beneficial vehicle for researchers to compare several solvents in analytical practices. As displayed in Fig. 4b, water achieves a G score of 7.3 with a greener and larger sphere than methanol, which attained a G score of 5.8 with a yellow and smaller sphere. This corresponds to approximately 125% enhancement in the greenness profile of water compared to methanol. This reveals the sustainability and eco-friendliness of water as a diluting solvent. Accordingly, water was selected as the optimal diluting solvent.

The impact of organized media on the fluorescence was explored, including different surfactants above their critical micellar concentrations and macromolecules. The study involved 1.0 mL of 2% w/v of each of the following: an anionic surfactant (SDS and CMC), a cationic surfactant (cetrimide), a molecular host (β-CD), and 1.0 mL of 2% v/v of nonionic surfactants (Tween 80 and Brij 35). Significant increases in fluorescence intensity were achieved using SDS as an enhancer and water as a diluting solvent, particularly for TLD, which reached up to five-fold enhancement (Fig. 4c). This increase in fluorescence intensity was ascribed to the electrostatic attraction between SDS (as an anionic surfactant), and TLD, DXZ, and TRZ, (as cationic targets), leads to deep insertion of targets in the micellar cores. This complexation imparts some rigidity to the targets and limits their free rotation, thereby reducing the likelihood of non-radiative processes. Additionally, employing SDS creates a viscous solution and enhances the chemical shielding around the targeted drugs, protecting the studied molecules from collisions, which decreases energy loss through non-radiative decay^9,45^. The volume of the SDS solution was thereafter examined for both mixtures using incremental increases from 0.1 to 2.0 mL, with maximum and plateau readings achieved between 1.5 and 1.7 mL, so 1.6 mL identified as optimal for consistent and maximum enhancement for all studied drugs, with 915%, 209%, and 133% sensitivity augmentation for TLD, DXZ, and TRZ, respectively. The pronounced fluorescence augmentation in TLD compared to DXZ and TRZ is related to its greater intrinsic hydrophobicity and smaller molecular size, with fewer polar functional moieties. These collectively enable TLD to penetrate deeper into the hydrophobic micellar cores and reduce external quenching. On the other side, the greater fluorescence enhancement of DXZ over TRZ was ascribed to the more hydrophobicity of the benzodioxane moiety (in DXZ), over the more hydrophilic tetrahydrofuran ring (in TRZ), which allows DXZ to more deeply integrate in the micellar core.

#### Impact of pH

The impact of pH on the SFI was assessed within the SDS system utilizing a 0.04 M BRB across a pH range of 2.3 to 12. As shown in Fig. 4d, the SFI of TLD remained stable up to pH 10, and it declined under strongly alkaline conditions. This reduction in SFI for TLD, which reached up to 60%, can be attributed to two potential factors. Firstly, at elevated pH levels, the phenolic groups in TLD are deprotonated, which in turn may lead to fluorescence quenching. Secondly, photo-induced electron transfer (PET) phenomena might occur in strongly alkaline conditions. At pH 12, the lone pair of the aliphatic tertiary amino group, which acts as a donor, transfers to the phenolic fluorophore, resulting in fluorescence inhibition. The PET process is not present at lower pH levels, where the lone pair is protonated and unavailable for transfer. Similar results were observed for DXZ and TRZ; there was no significant increase in low pH values, and a decrease was observed at high pH values. The activation of the PET process also accounts for the reduced SFI of DXZ and TRZ at pH levels above 8, reaching approximately 29% for both drugs. This occurs as the lone pair from the piperazine ring is transferred to the quinazoline fluorophore, leading to fluorescence quenching for both drugs. Based on these findings, no buffer was used in the study, which streamlined the proposed procedures.

### Validation criteria

The developed methodology was validated according to the International Council for Harmonization (ICH) guidelines^46^ as follows:

Under optimal conditions, the constructed calibration graphs showed linear correlations over a wide nanoscale concentration range of (20.0–200.0), (5.0–50.0), and (5.0–50.0) ng mL⁻^1^ for TLD, DXZ, and TRZ, respectively. The closeness of the computed correlation coefficients to unity validated excellent linear relationships (Table 1). The limit of detection (LOD) and limit of quantification (LOQ) were calculated for each analyte as sensitivity measures. LOD was determined as three times the division of the SD of the intercept by the slope, while LOQ is ten times the same ratio. Utilizing the SDS enhancer improves the spectrofluorimetric sensitivity of each analyte, achieving LODs of 1.25, 0.90, and 0.73 ng mL^−1^, with LOQs of 3.78, 2.73, and 2.20 ng mL^−1^ for TLD, DXZ, and TRZ, respectively. This exceptional nano-level sensitivity enabled the detection of the studied targets in spiked human plasma with satisfactory accuracy.

**Table 1:** Performance data for assessing TLD, DXZ, and TRZ by the proposed method.

Parameter	TLD	DXZ	TRZ
Wavelength of detection (nm)	292	355	355
Concentration range (ng mL^−1^)	20.00–200.00	5.00–50.00	5.00–50.00
LOD (ng mL^−1^)	1.25	0.90	0.73
LOQ (ng mL^−1^)	3.78	2.73	2.20
Correlation coefficient (r)	0.9999	0.9997	0.9998
Slope	0.12	1.03	1.25
Intercept	2.14	0.41	2.32
S_y/x_	0.06	0.40	0.39
S_a_	0.04	0.28	0.27
S_b_	0.0003	0.01	0.01
%Error	0.21	0.48	0.49
%RSD	0.56	1.37	1.38
No. of experiments	7	8	8
Mean found (%) ± SD	99.94 ± 0.56	99.74 ± 1.37	99.74 ± 1.38

N.B. S_y/x_ = standard deviation of the residuals. S_a_ = standard deviation of the intercept of the regression line. S_b_ = standard deviation of the slope of the regression line. %Error = RSD% $\sqrt{n}$

Precision was evaluated through analysis at three concentration levels spanning the linearity range for each drug. The study included the analysis of (40.0, 100.0, and 120.0) ng mL^−1^ for TLD and (15.0, 25.0, and 40.0) ng mL^−1^ for both DXZ and TRZ. These concentrations were analyzed on three successive occasions within one day to assess repeatability (intraday precision) and over subsequent days to evaluate reproducibility (intermediate precision) (Table S1). Tolerable %RSD levels were obtained for each target, indicating satisfactory precision.

Accuracy was verified through the analysis of seven concentration levels for TLD and eight levels for DXZ and TRZ in their raw material within the linearity ranges, achieving high %recoveries with low %Errors for all studied drugs. Furthermore, the results were statistically compared with published works using Student’s *t-*test and the variance ratio* F*-test^47^. As indicated in Table 2, there was no significant discrepancy between the overall results, assuring the accuracy and precision of the proposed method.

**Table 2:** Application of the proposed and reported methods for determining TLD, DXZ and TRZ in their raw material.

Compound	Amount taken (ng mL^−1^)	Amount found (ng mL^−1^)	%Found	Reported methods^9,19,30^
TLD	20.00	19.959	99.80	101.22
	40.00	39.562	98.91	98.97
	60.00	60.381	100.64	100.42
	100.00	100.191	100.19	
	120.00	120.446	100.37	
	160.00	159.459	99.66	
	200.00	199.974	99.99	
$\overline{x }$± SD			99.94 ± 0.56	100.20 ± 1.14
Student’s *t-*test			0.29 (2.31)*	
Variance ratio (*F* test)			4.14 (5.14)*	
DXZ	5.00	4.955	99.10	99.00
	10.00	9.817	98.17	101.13
	15.00	14.867	99.11	99.58
	20.00	19.728	98.64	
	25.00	25.449	101.80	
	30.00	30.407	101.36	
	40.00	40.322	100.81	
	50.00	49.465	98.93	
$\overline{x }$± SD			99.74 ± 1.37	99.90 ± 1.10
Student’s *t-*test			0.20 (2.26)*	
Variance ratio (*F* test)			1.55 (19.35)*	
TRZ	5.00	4.856	97.12	101.63
	10.00	9.949	99.49	97.90
	15.00	14.924	99.49	100.22
	20.00	20.120	100.60	
	25.00	25.398	101.59	
	30.00	30.228	100.76	
	40.00	39.495	98.74	
	50.00	50.062	100.12	
$\overline{x }$± SD			99.74 ± 1.38	99.92 ± 1.88
Student’s *t-*test			0.15 (2.26)*	
Variance ratio (*F* test)			1.86 (4.73)*	

*Values between parentheses are the tabulated* t* and *F* values, respectively, at p = 0.05 ^47^.

The specificity study was conducted to assess the practicability of the proposed procedures for measuring the targeted analytes in the presence of various interferents. The method was followed to assess TLD, DXZ, and TRZ in their individual dosage forms. The satisfactory outcomes demonstrated the capacity of the prescribed method to detect the studied drugs in their pharmaceutical dosage forms without significant interference from tablet excipients. Additionally, selectivity was assessed by evaluating each drug in its laboratory-prepared binary mixture. The results confirmed the feasibility of the proposed methodology for the selective quantification of each drug without mutual interference. In terms of biological application, the suggested method was appraised to quantify the studied analytes in spiked human plasma. Plausible %recoveries and %RSD values were obtained, reflecting the reliability, specificity, and selectivity of the proposed work. Finally, tap and river water matrices were utilized to assess the specific adequacy of the method for detecting the studied drugs in environmental samples. The achieved satisfactory percentage recoveries revealed the reliable capability of the designed method for recording the studied analytes in environmental water samples without interference. The obtained results collectively declare the specificity and selectivity of the introduced framework toward recording TLD, DXZ and TRZ in assorted matrices. The suggested work was subjected to one-way ANOVA for a rigorous comparison of the studied matrices, including plasma, tap and river water. As represented in Table S2, no significant differences were observed between the different matrices for all targeted analytes.

Additionally, the matrix effect (ME%) was evaluated at 100.0 ng mL⁻^1^ for TLD, and at 30.0 ng mL⁻^1^ for both DXZ and TRZ, using the following formula^48^:

$$ME\% \, = \left( {{{\left( {F2 - F1} \right)} \mathord{\left/ {\vphantom {{\left( {F2 - F1} \right)} {F1}}} \right. \kern-0pt} {F1}}} \right) \times 100$$

Where F1 represents the analyte’s signal in the matrix-free standard, and F2 represents the analyte’s signal in the matrix-matched standard.

As depicted in Table S3, a significant plasma matrix effect was noted for all targeted analytes, with a suppression trend indicated by ME% ≥ -15, which was compensated by implementing matrix-matched calibration^48,49^. However, no significant effect was observed for either river or tap water regarding the studied analytes.

These outputs jointly manifest that the prescribed framework complies with validation requirements outlined in the ICH standards.

### Multifunctional applications of the proposed methodology

#### Laboratory-synthesized mixtures

**Fig. 5:**
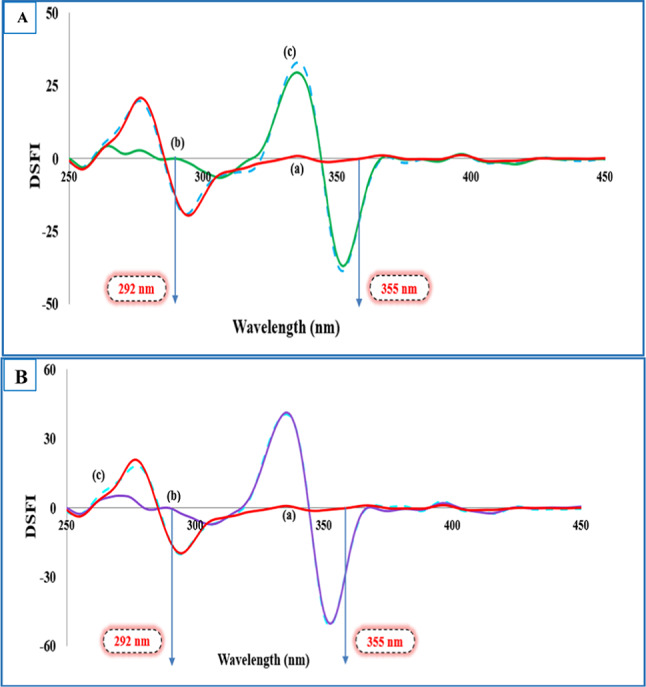
(**A**) First derivative synchronous fluorescence spectra at Δλ = 20 nm of: (**a**) TLD 120.0 ng mL⁻^1^. (**b**) DXZ 30.0 ng mL⁻^1^. (**c**) Synthetic mixture of TLD 120.0 ng mL⁻^1^ and DXZ 30.0 ng mL⁻^1^. (**B**) First derivative synchronous fluorescence spectra at Δλ = 20 nm of: (**a**) TLD 120.0 ng mL⁻^1^. (**b**) TRZ 30.0 ng mL⁻^1^. (**c**) Synthetic mixture of TLD 120.0 ng mL⁻^1^ and TRZ 30.0 ng mL⁻^1^.

The published medical reports advocate for a combination therapy of TLD and DXZ in a 1:1 ratio to alleviate urinary tract symptoms in patients suffering from benign prostatic hyperplasia^1^. For complications related to ureteral stents, evidence suggests that administering TLD alongside TRZ at a 1:2 ratio proves effective^2^. Considering these facts, four laboratory-prepared mixtures of the studied binary mixtures were created by blending various ratios of the cited medications, including their prescribed combination therapy ratio and ratios of their available dosage forms (Fig. 5a, b). The abridged results in Table S4 demonstrated the acceptable percentage recoveries achieved for the studied drugs without mutual interference. This warranted the selective surveillance of the proposed platform for assessing the targeted drugs in each binary mixture.

#### Pharmaceutical dosage forms

The suitability of the proposed method was assessed for quantifying TLD, DXZ, and TRZ in their commercial formulations, such as Incont L. A^®^ (4 mg/ tablet), Dosin^®^ (1 mg/ tablet), and Itrin^®^ (2 mg/ tablet), respectively. The percentage recoveries, along with their SD, were computed for each analyte and presented in Table 3. These results were closely aligned with the label claims of the targeted drugs, without intrusion from pharmaceutical excipients. Furthermore, published studies were referenced to verify the recoveries of the examined analytes in the same dosage forms. For TLD, the comparison method involved monitoring its fluorescence intensity at λ_ex/em_ of 282/310 nm, using 1.5 mL of 1% w/v SDS and water as diluting solvents^9^. In the case of DXZ, its absorption spectrum was directly measured at 245 nm using 0.1 N HCl as the diluting solvent^19^. For TRZ, the fourth derivative of the absorption spectra was recorded at 329.2 nm employing (∆λ = 4 nm), a scaling factor of 100, and diluting with methanol^30^. Statistical comparisons between the results obtained from published methodologies and those developed in this study were performed using Student’s *t-*test and the variance ratio *F-*test^47^. The findings listed in Table 3 indicated no discernible difference between the proposed method and the published works in terms of accuracy and precision^47^.

**Table 3:** Application of the proposed and reported methods for assessing TLD, DXZ, and TRZ in pharmaceutical dosage forms.

Compound	Amount taken (ng mL^−1^)	Amount found (ng mL^−1^)	%Found	Reported methods^9,19,30^
TLD Incot L. A^®^ tablets (4 mg/ tablet)	40.00	38.804	97.01	98.67
	100.00	97.970	97.97	98.51
	120.00	117.972	98.31	99.58
	160.00	159.632	99.77	
$\overline{x }$± SD			98.27 ± 1.14	98.92 ± 0.58
Student’s *t-*test			0.99 (2.57)*	
Variance ratio (*F* test)			3.86 (19.16)*	
DXZ Dosin^®^ tablets (1 mg/ tablet)	10.0	9.918	99.18	98.65
	20.0	20.096	100.48	101.25
	30.00	29.649	98.83	99.52
	40.00	40.720	101.80	
$\overline{x }$± SD			100.07 ± 1.35	99.81 ± 1.32
Student’s *t-*test			0.25 (2.57)*	
Variance ratio (*F* test)			1.05 (19.16)*	
TRZ Itrin^®^ tablets (2 mg/ tablet)	10.0	9.781	97.81	101.23
	20.0	19.646	98.23	98.16
	30.00	29.646	98.82	100.20
	40.00	39.748	99.37	
$\overline{x }$± SD			98.56 ± 0.68	99.86 ± 1.56
Student’s *t-*test			1.35 (2.57)*	
Variance ratio (*F* test)			5.26 (9.55)*	

*Values between parentheses are the tabulated* t* and *F* values, respectively, at p = 0.05 ^47^.

The ultra-sensitive, user-friendly, eco-friendly, and accurate nature of the proposed method highlights its reliability in tracking TLD, DXZ, and TRZ in their pharmaceutical dosage forms. The outcomes also showcase the value of the prescribed systems as an evaluation tool for the nominated drugs in the quality control of pharmaceutical forms.

#### Checking content uniformity

The notable attributes of the introduced system, including superb sensitivity, facility and short analysis time, guarantee the design to be a suitable tool for checking content uniformity in the target’s dosage forms. Content uniformity is a crucial quality control test that checks the consistency of the active ingredient in each individual tablet or capsule and ensures it is within an acceptable limit. The low concentration of TLD, DXZ, and TRZ in Incont L. A^®^ (4 mg/ tablet), Dosin^®^ (1 mg/ tablet), and Itrin^®^ (2 mg/ tablet), respectively, entails an accurate methodology to test their uniformity. The study was carried out following ICH guidelines^42^. The computed acceptance values were 5.38, 5.35, and 4.85 for TLD, DXZ, and TRZ, respectively, which were all below the maximum allowed acceptance value (15) (Table S5). These outcomes reveal the uniformity of the targeted analytes in the tested formulations.

#### Biological samples

Reported pharmacokinetic data for TLD, DXZ, and TRZ confirmed that their plasma levels fall within the method linearity ranges^50^. The impressive ultra-sensitivity of the designed framework enabled the accurate scaling of the analyzed drugs. To assess the specificity and selectivity of the designed systems toward the drugs’ signal in the complex plasma matrix, analysis of each binary mixture in plasma samples was conducted. Matrix-matched calibration for each drug in both studied mixtures was executed to determine the percentage recoveries and SD values. As shown in Table S6, the results indicated acceptable percentage recoveries with SD values not exceeding 2.83, 1.70 and 0.83 for TLD, DXZ, and TRZ, respectively. Notably, the findings indicated that simple plasma protein precipitation procedures were adequate to eliminate the potential interference that may arise from the existing lipids, proteins, and endogenous components in the plasma matrix. The proposed protocol, with its facility, affordability, sensitivity, and energy-saving characteristics, collectively guarantees its applicability as a quantitative candidate for monitoring the investigated medications in biological fluids.

#### Environmental water samples

Due to increasing contamination of water by pharmaceutical residues, the proposed method was applied to tap water and the River Nile water. Matrix-matched calibrations were performed, yielding satisfactory recoveries for the studied drugs with SD values that didn’t exceed 0.66, 0.89 and 0.92 for TLD, DXZ and TRZ in tap water or 1.30, 1.57 and 0.59 for TLD, DXZ and TRZ in river water (Table S7). These findings settled the specificity of the developed systems for monitoring TLD, DXZ, and TRZ in environmental water samples without significant interference. Remarkably, the water samples were directly analyzed without extensive extraction processes or complicated instrumentation. These sensitive and streamlined procedures render the proposed work a suitable indicator for monitoring water contamination and environmental testing. Additionally, the fast response scanning using minimal sample size, with only filtration as a pretreatment step, represents the introduced work as an innovative step toward point-of-care water testing.

### Greenness profile

Over the last decade, GAC has gained significant recognition in both research and industrial applications. GAC advocates for the reduction of hazards produced by analytical procedures, focusing on protecting human health and minimizing environmental impact. The framework proposed here aims to prioritize GAC principles by utilizing biodegradable chemicals and environmentally friendly solvents in analytical processes. Consequently, a plethora of newly introduced assessment tools have been employed in this context to evaluate the suggested analytical systems and provide a holistic view of their environmental friendliness. In this context, the Modified Green Analytical Procedure Index (MoGAPI) was executed using convenient software^51^. The results are depicted in a pentagram format, with colors indicating impact: green, yellow, and red corresponding to favorable, moderate, and unfavorable environmental impact, respectively, with an overall score^52^. The proposed methodology and the reported spectrofluorimetric method^9^ received the highest number of green sections and a total score of 88. This advantage is related to exploiting SDS as a biodegradable surfactant and water as an eco-friendly diluent, which are the key merits of the designed approaches. However, the assessment by MoGAPI does not consider the procedure’s speed and complex steps. Therefore, the Analytical Greenness Metric Approach (AGREE) was also employed to obtain a thorough appraisal. The evaluation in AGREE revolves around the twelve fundamentals of GAC, and the findings are displayed *via* a clock-inspired visual representation, with twelve sections colored using a traffic-light color-coding system^53^. The center of the pictogram achieves an overall average color with a score scaled up to one^54,55^. As demonstrated in Table 4, the introduced system, along with the reported methodologies^9,23^, achieved the highest score of 0.76, reflecting the largest number of green sections. The excellence of the prescribed framework is attributed to the reliance on derivatization-free assaying procedures using the eco-friendly SDS fluorescent enhancer and water as a diluting solvent. This stands in stark contrast to previously reported methods that employed hazardous chemicals^8,22^ (such as dansyl chloride, dichloromethane, mercaptoethanol, and orthophthalaldehyde) and involved lengthy procedures lasting up to 45 minutes^22^ or requiring heating for 10 min at 40 °C^8^.

**Table 4:**
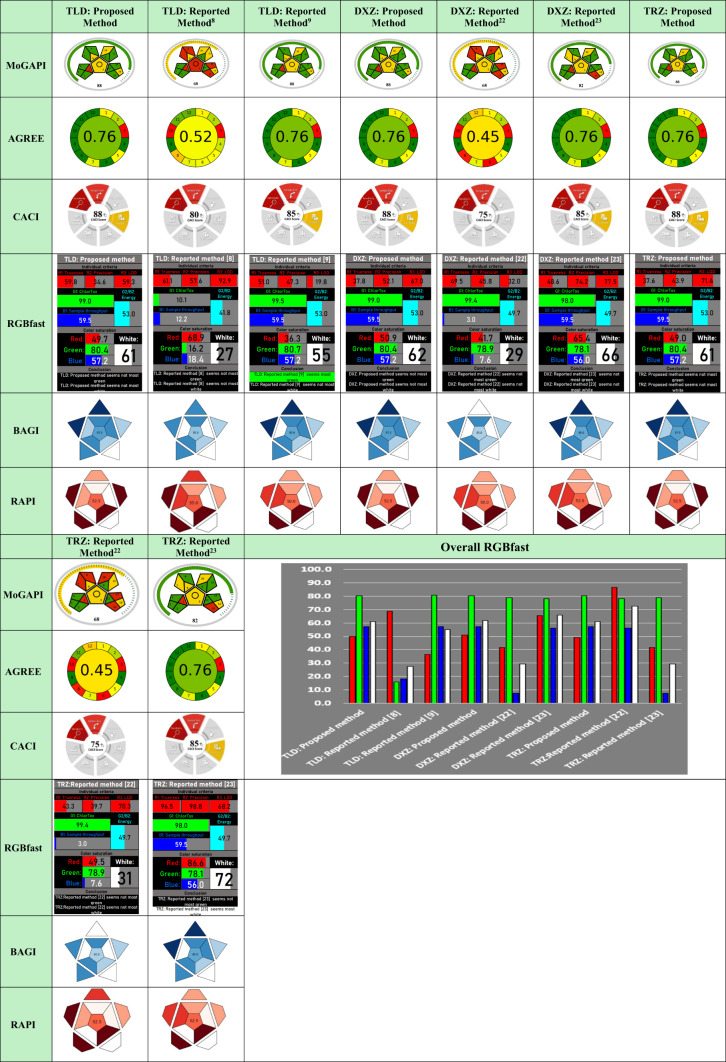
Assessment of the suggested and the reported spectroscopic methods for TLD, DXZ, and TRZ using MoGAPI, AGREE, CACI, RGBfast, RAPI and BAGI.

All green metrics applied supported the suggested protocol’s minimal negative impacts on the environment and human health.

### Whiteness profile

In 2021, a recent perspective was introduced as an extension of GAC, nominated as white analytical chemistry (WAC)^56^. It aims to offer energy and cost-effective, swift, and straightforward analytical methodologies, ushering in a paradigm shift. This comprehensive approach is grounded in twelve fundamental standards, propelling it beyond the limitations of traditional GAC. In the suggested work, the fast red green blue 12 model (RGBfast) was executed for a multifaceted assessment framework^57^. The red criteria evaluate the method’s efficiency and validation parameters. For the green criteria, the focus is on the ethos of GAC, examining sustainability and ecological factors. Meanwhile, the blue criteria reflect affordability, speed, and ease of use. The whiteness attribute was assessed using an Excel sheet and presents the findings as a mean value ranging from 0 to 100 and a corresponding color scale extending from black to white^58^. A key hallmark of the RGBfast model, compared to traditional RGB models, is its algorithmic support and independence from personal bias, providing a more objective evaluation of the whiteness of the analytical methods^57^. In addition to integrating the ChlorTox scale, which not only evaluates solvent toxicity but also integrates multiple green chemistry principles consistent with GAC. As depicted in Table 4, the proposed methodology achieved acceptable whiteness scores of 61 for TLD and TRZ, and 62 for DXZ, respectively. These results confirm adherence to the whiteness postulates as a significant step toward sustainable analytical performance.

### Redness profile

The Red Analytical Performance Index (RAPI) is an evaluation tool designed to assess analytical performance in terms of validation parameters of LOQ, range precision and matrix effect^59^. RAPI relies on the principles of WAC together with the red criteria of the RGB model to systematically estimate these parameters. The evaluation is delivered through a star-shaped pictogram colored with gradient red shades, and an overall numerical score from 25 to 100. As represented in Table 4, the proposed methodology achieved a reasonable score of 52.5, revealing acceptable analytical performance.

### Blueness profile

Regarding the assessment of the blueness attribute, the Blue Applicability Grade Index (BAGI) was employed to ensure a comprehensive sustainability evaluation. The blueness measure incorporates ten practical factors to determine the suitability and applicability of the analytical framework for practical use. BAGI utilizes an open-source application and a web link to present outcomes in a steroid graph featuring blue shade gradients that indicate varying levels of compliance with practical attributes. Additionally, each assessment receives an overall score between 25 and 100, displayed at the center of the graph^60^. BAGI’s evaluation offers quick and insightful qualitative and quantitative assessments, facilitating the comparison of various methods in practical settings while highlighting the strengths and weaknesses of each system^61,62^.

The data shown in Table 4 demonstrates the enhanced performance of the proposed framework over the previously reported methods, evidenced by a score of 67.5 for the proposed methodologies, while 65 for methods^9,23^, 55 for method^8^ and 50 for method^22^.

Additionally, the Click Analytical Chemistry Index (CACI) assessment tool was employed to evaluate the practicality and applicability of the designed method^63^. CACI considers factors such as sample size, preparation, feasibility, applicability, portability, sensitivity, and automation. The evaluation is presented through colored pictograms, where colored, grey and black represent excellent, intermediate, and poor performance, respectively. As represented in Table 4, the suggested method outperformed the reported work, achieving scores of 88, compared to scores of 85 for methods^9,23^, while 80 and 75 for method^8^ and method^22^, respectively.

This success is ascribed to the straightforward proposed steps, which rely on a direct-mix-and-read assay in a micellar system, eliminating the need for derivatizing agents, thereby enhancing productivity. In contrast, published spectrofluorimetric methods relied on derivatization reactions with chemicals that may not be available in standard labs. This, in addition to the lengthy procedures, compromises practicality and throughput.

### GLANCE overview

**Fig. 6:**
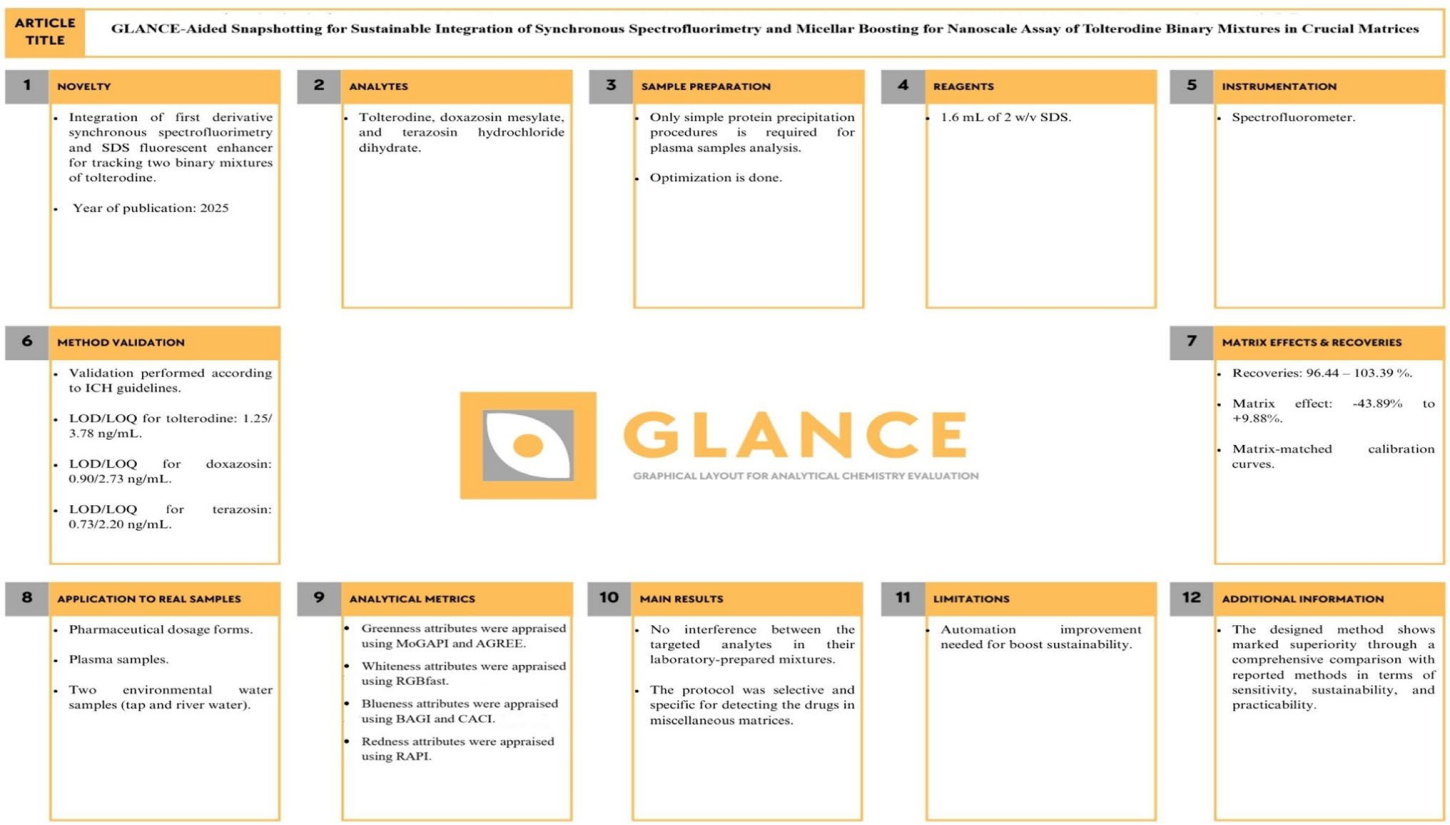
GLANCE layout for analysis of two binary mixtures of TLD.

The Graphical Layout Tool for Analytical Chemistry Evaluation (GLANCE) was designed by Adrian Fuente-Ballesteros *et. al*^64^, is a free andeditable template dedicated to giving a visual summary of the analytical chemistry methodology in a concise and well-organized tabulated format . The foundation of GLANCE relies on twelve aspects, as follows (Fig. 6):

1. *Novelty:* points out the unique facets of the method, as sensitivity, scope of analysis, and sustainability.
2. *Analytes:* displays the targeted analytes.
3. *Sample preparation:* mentions the time/steps required for the preparation process.
4. *Reagents:* presents the utilized chemicals and reagents in the analysis process.
5. *Instrumentation:* detail the analytical tools employed through the analytical method.
6. *Method validation:* reveals whether the method is validated and expresses LOD and LOQ values.
7. *Matrix effects and recovery:* draws attention to the matrix effects on analytes’ signals.
8. *Application to real samples:* reports the type of reliable analytical application in the proposed method.
9. *Analytical metrics:* addresses the employed greenness and blueness evaluation tools in the suggested work.
10. *Main results:* outlines a summary of the key results.
11. *Limitations:* highlights the constraints observed for the described methodology.
12. *Additional information:* nominates any additional information, precautions, and protocols necessary for the analysis process.

### The introduced method *versus *different works for recording the studied analytes

The proposed framework for monitoring TLD, DXZ, and TRZ has shown greater analytical efficiency compared to the previous approaches. As illustrated in Table 5, the developed protocol demonstrated excellent sensitivity levels, ranging from 5 to 50 times and 4 to 109.4 × 10^3^ times, and 2 to 1000 times more sensitive than previously published methods for detecting TLD, DXZ, and TRZ, respectively. Although techniques such as HPLC–MS/MS^32^ and voltammetry^33^ offer improved sensitivity for TRZ detection, they require skilled operators and are often inaccessible in most laboratories. Additionally, they involve a multistep analysis that slows down the process, which decreases throughput. The ecological profile of the proposed method is also advantageous, as conventional detection methods rely on hazardous reagents like acetonitrile and triethylamine, besides generating significant waste during the chromatographic analytical process. The spectrofluorimetric methods utilize orthophthalaldehyde, 2-mercaptoethanol^22^, dansyl chloride, and dichloromethane^8^, which are harmful to aquatic life. In potentiometric methods, the analysis relies on N-bromosuccinimide and N-bromophthalimide^20^, both of which are carcinogenic and potentially lead to genetic defects. In contrast, the proposed approach boosts the value of the environmentally friendly SDS and water, which are viable for analytical practices in resource-limited laboratories. The streamlined procedures are another point of efficiency where the traditional chromatographic and capillary electrophoresis methods are often complicated by requiring preconditioning steps and preparation of mobile phases or background electrolytes. Conversely, the suggested method depended on a direct-mix-and-read assay to detect the two targets in each binary mixture. While some reported methods demonstrate high sensitivity, their lengthy protocols, such as heating at 40 °C for 10 minutes^8^ or waiting for 45 minutes^22^, can reduce throughput. Furthermore, the wide analytical scope represents a key advantage; as shown in Table 5, many reported methods are restricted to pharmaceutical and/or biological applications, while the proposed method extends their applicability to environmental studies in addition to pharmaceutical and biological matrices. Collectively, these features confirm that a single experimental setup can address all the challenges mentioned, eliminating time-consuming separation steps, complex instruments, and hazardous chemicals while maintaining adequate sensitivity for detecting targeted analytes in various matrices. This positions the proposed work as a benchmark tool for the nominated targets in quality control laboratories, therapeutic drug monitoring, and environmental pollution control.

**Table 5:** Comparison between different published works for tracking TLD, DXZ, and TRZ with the developed methodology.

	Method	Correlation coefficient	Linearity	LOD	Conditions	Matrix	Refs.
TLD	HPLC-fluorescence detection	0.9997	1.0–15.0 µg mL^−1^	0.30 µg mL^−1^	C_18_ column, gradient mobile phase consisting of acetonitrile, water, and phosphate buffer of pH 3.0, fluorescence detection λ_ex/em_ of 280/350 nm, achieving elution within 7.26 min	Dosage forms and plasma	^12^
	HPLC–UV	0.9991	0.1–100.0 µg mL^−1^	0.016 µg mL^−1^	C_18_ column, mobile phase consisting of acetonitrile: 0.05 M pentane sulfonic acid sodium salt of pH 5.5 (50:50, v/v), UV at 200 nm, achieving elution within 5.8 min	Dosage forms	^13^
	Capillary electrophoresis	0.9976 for R enantiomer 0.9978 for S enantiomer	10.0–300.0 ng mL^−1^	3.0 ng mL^−1^	Background electrolytes consist of a 100 mM phosphate running buffer of pH 4.0, containing 20% (w/v) maltodextrin with dextrose as chiral selector, applied voltage of 20 kV and 20 °C, achieving elution for R and S enantiomers within 26.3 and 26.8 min, respectively	Plasma and Urine	^14^
	Voltammetry	0.9994	2.0–12.5 μmol L^−1^	0.43 µmol L^−1^	Cyclic voltametric determination using ion transfer voltammetry at a polarized room temperature ionic liquid membrane, with and potential pulse width of 0.2 s, a sampling time of 0.02 s, and a pulse period of 0.5 s	Urine	^16^
	Spectrophotometry	0.9999	1.0–10.0 µg mL^−1^	0.10 µg mL^−1^	Formation of ion pair complex with eosin at pH 3.8 and measurement of the absorbance immediately at 545 nm	Dosage forms	^6^
	Spectrofluorimetry	0.9981	5.0–60.0 ng mL^−1^	0.14 ng mL^−1^	Derivatization was performed with dansyl chloride at pH 9.5 to yield a highly fluorescent derivative. The reaction mixture was incubated at 40 °C for 10 min, followed by extraction with dichloromethane. Detection was carried out at λ_ex/em_ 360/590 nm	Dosage forms and plasma	^8^
	Spectrofluorimetry	0.9998	25.0–500.0 ng mL^−1^	7.37 ng mL^−1^	Micellar enhancement was achieved using SDS, and the fluorescence was measured immediately at λ_ex/em_ 282/310 nm	Dosage forms and plasma	^9^
	This work	0.9999	20.0–200.0 ng mL^−1^	1.24 ng mL^−1^	First-derivative synchronous spectrofluorimetry was performed with micellar enhancement using SDS, and the fluorescence was measured immediately at 292 nm	Dosage forms, environmental water, and plasma	
DXZ	HPLC–UV	0.9998	2.50–25.0 µg mL^−1^	0.28 µg mL^−1^	C_18_ column, mobile phase consisting of acetonitrile: 50 mM phosphate buffer (30:70, v/v) adjusted at pH 3, UV detection at 230 nm, achieving elution within 8 min	Dosage forms	^26^
	HPLC–UV	0.9998	24.0–290.0 µg mL^−1^	1.75 µg mL^−1^	C_18_ column, mobile phase consisting of 25 mM ammonium acetate of pH 4: acetonitrile (50:50, v/v), PDA detection at 230 nm, achieving elution within 8 min	Dosage forms	^27^
	Capillary electrophoresis	0.9999	50.0–2000.0 ng mL^−1^	9.75 ng mL^−1^	Field amplified sample injection combined with micellar electrokinetic chromatography using 30 mM SDS and 8% butanol, applying a voltage of 28 kV at 25 °C, achieving elution within 5 min	Dosage forms	^28^
	Potentiometry	NA	0.547–2.188 mg	NA	Titration of DXZ against N-bromosuccinimide or N-bromophthalimide in an acidic medium, and the endpoint is determined potentiometrically using a platinum electrode	Dosage forms	^20^
	Spectrophotometry	0.9996µg mL^−1^ for bromocresol green 0.9995 µg mL^−1^ for phenol red	1.0–19.0 µg mL^−1^ for bromocresol green 2.0–21.0 µg mL^−1^ for phenol red	0.18 µg mL^−1^ for bromocresol green 0.31 µg mL^−1^ for phenol red	Formation of ion pair complex with bromocresol green or phenol red, and the absorbance was measured immediately at 418 and 420 nm, respectively	Dosage forms	^20^
	Spectrofluorimetry	0.9994	20.0–400.0 ng mL^−1^	3.88 ng mL^−1^	Derivatization with orthophthalaldehyde in the presence of 2-mercaptoethanol in borate buffer (pH 9.7) was carried out to generate a highly fluorescent isoindole derivative after 45 min of incubation, which was detected at λ_ex/em_ 337/430 nm	Dosage forms and plasma	^22^
	Spectrofluorimetry	0.9999	2.0–60.0 ng mL^−1^	0.53 ng mL^−1^	Micellar enhancement was achieved using polyoxyethylene 50 stearate, and the fluorescence was immediately measured at λ_ex/em_ 340/382 nm	Dosage forms and plasma	^23^
	This work	0.9997	5.0–50.0 ng mL^−1^	0.90 ng mL^−1^	First-derivative synchronous spectrofluorimetry was performed with micellar enhancement using SDS, and the fluorescence was measured immediately at 355 nm	Dosage forms, environmental water, and plasma	
TRZ	HPLC–UV	0.9998	2.50–25.0 µg mL^−1^	0.23 µg mL^−1^	C_18_ column, mobile phase consisting of acetonitrile: 50 mM phosphate buffer (30:70, v/v) adjusted at pH 3, UV detection at 230 nm, achieving elution within 8 min	Dosage forms	^26^
	HPLC–UV	0.9998	5.0–80.0 µg mL^−1^	NA	C_18_ column, mobile phase consisting of acetonitrile: methanol: water: triethylamine (pH 5.6), in the ratio of (45: 45: 10: 0.2, by volume) at ambient temperature, UV detection at 225 nm, achieving elution within 4 min	Dosage forms	^30^
	HPLC–MS/MS	> 0.99	1.0–100.0 ng mL^−1^	NA	C_18_ column, mobile phase consisting of acetonitrile: 0.1% (v/v) formic acid (70:30, v/v) with MS/MS detection, achieving elution within 6 min	Dosage forms and plasma	^32^
	Capillary Electrophoresis	0.9999	50.0–2000.0 ng mL^−1^	9.04 ng mL^−1^	Field amplified sample injection combined with micellar electrokinetic chromatography using 30 mM SDS and 8% butanol, applying a voltage of 28 kV at 25 °C, achieving elution within 5 min	Dosage forms	^28^
	Voltammetry	0.997 for 60 s 0.998 for 300 s	8.0 × 10^−9^ to 1.0 × 10^−7^ M for 60 s 1.0 × 10^−9^ to 3.0 × 10^−8^ M for 300 s	2.0 × 10^−10^ M for 60 s 1.5 × 10^−11^ M for 300 s	Square-wave adsorptive cathodic stripping voltammetry, the peak current was measured within accumulation times of 60 s and 300 s	Dosage forms and serum	^33^
	Spectrophotometry	0.997 for the fourth derivative method 0.998 for the derivative ratio method	2.0–22.0 µg mL^−1^ for the fourth derivative method 2.0–20.0 µg mL^−1^ for the derivative ratio method	NA	The fourth-derivative spectrophotometric method employing (∆λ = 4 nm), a scaling factor of 100, and the absorbance was measured immediately at 329.2 using methanol as a solvent. The first derivative of the ratio spectra employing (∆λ = 4 nm), a scaling factor of 10, and the absorbance was measured at 310.6 nm using methanol as a solvent	Dosage forms	^30^
	Spectrofluorimetry	0.9989	10.0–300.0 ng mL^−1^	0.77 ng mL^−1^	Derivatization with orthophthalaldehyde in the presence of 2-mercaptoethanol in borate buffer (pH 9.7) was carried out to generate a highly fluorescent isoindole derivative after 45 min of incubation, which was detected at λ_ex/em_ 337/430 nm	Dosage forms and plasma	^22^
	Spectrofluorimetry	0.9999	4.0–100.0 ng mL^−1^	0.85 ng mL^−1^	Micellar enhancement was achieved using polyoxyethylene 50 stearate, and the fluorescence was immediately measured at λex/em 340/382 nm	Dosage forms and plasma	^23^
	This work	0.9998	5.0–50.0 ng mL^−1^	0.73 ng mL^−1^	First-derivative synchronous spectrofluorimetry was performed with micellar enhancement using SDS, and the fluorescence was measured immediately at 355 nm	Dosage forms, environmental water, and plasma	

## Conclusion

The integration of CWSS with micellar augmentation has enabled the development of a derivatization-free strategy for ultra-trace tracking of TLD in the presence of DXZ or TRZ through discriminated matrices. The prescribed streamlined procedures efficiently evaluated the cited drugs in raw materials, laboratory-prepared mixtures, and pharmaceutical preparations with tolerable recoveries. The clinically relevant sensitivity allowed surveillance of targeted drugs in human plasma. Moreover, the research was expanded to investigate the method’s specificity in two environmental water samples and to test the matrix impediments, while recognizing the limited number of samples analyzed. The introduced work excelled over the reported methods regarding sensitivity, affordability, short analysis time, and adherence to sustainability postulates. The recently introduced GLANCE tool was used to display the work’s features and attributes without wading through detailed data. Greenness, sustainability and practicability profiles reveal the proficiency of the proposed work that can usher in a paradigm shift towards a sustainable community. By integrating sustainability and practicability principles with innovative design, the proposed strategy paves the way toward sustainable and point-of-care analytical solutions.

## Supplementary Information

Below is the link to the electronic supplementary material.

Supplementary Material 1

## Data Availability

Data will be made available on request.
