# Supplementary material for: GLANCE-aided snapshotting for sustainable integration of synchronous spectrofluorimetry and micellar boosting for nanoscale assay of tolterodine binary mixtures in crucial matrices
Source: Sci Rep. 2025 Dec 5;15:43197. doi: 10.1038/s41598-025-27144-0 (PMC12680775; doi:10.1038/s41598-025-27144-0)
Supplement: Supplementary file 1 — Supplementary Material 1 [file 41598_2025_27144_MOESM1_ESM.docx]

**GLANCE-Aided Snapshotting for Sustainable Integration of Synchronous Spectrofluorimetry and Micellar Boosting for Nanoscale Assay of Tolterodine Binary Mixtures in Crucial Matrices**

**Eman Yosrey*^1^, Heba Elmansi^1^,** [**Shereen**](https://analyticalsciencejournals.onlinelibrary.wiley.com/action/doSearch?ContribAuthorStored=Sheribah%2C+Zainab) **Shalan^1^, Jenny Jeehan Nasr^1^**

1. Department of Pharmaceutical Analytical Chemistry, Faculty of Pharmacy,

Mansoura University, Mansoura 35516, Egypt.

* Author to whom correspondence should be addressed.

E-mail address: [eman_yosrey55@mans.edu.eg](mailto:eman_yosrey55@mans.edu.eg), [emanyosrey435@gmail.com](mailto:emanyosrey435@gmail.com)

**Fig. S1:**

**A:** Native fluorescence spectra of:

(a, b) Excitation and emission of TLD (200.0 ng mL^-^¹) using methanol as a diluting solvent.

(c, d) Excitation and emission of DXZ (75.0 ng mL^-^¹) using methanol as a diluting solvent.

**B:** Native fluorescence spectra of:

(a, b) Excitation and emission of TLD (200.0 ng mL^-^¹) using methanol as a diluting solvent.

(c, d) Excitation and emission of TRZ (75.0 ng mL^-^¹) using methanol as a diluting solvent.

**Fig. S2:**

**A:** Synchronous fluorescence spectra at Δλ = 20 nm of:

(a) DXZ (5.0, 10.0, 15.0, 20.0, 25.0, 30.0, 40.0, 50.0) ng mL⁻¹.

(b) TLD 120.0 ng mL⁻¹.

**B:** Synchronous fluorescence spectra at Δλ = 20 nm of:

(a) TLD (20.0, 40.0, 60.0,100.0, 120.0, 160.0, 200.0) ng mL⁻¹.

(b) DXZ 20.0 ng mL⁻¹.

**Fig. S3:**

**A:** Synchronous fluorescence spectra at Δλ = 20 nm of:

(a) TRZ (5.0, 10.0, 15.0, 20.0, 25.0, 30.0, 40.0, 50.0) ng mL⁻¹.

(b) TLD 120.0 ng mL⁻¹.

**B:** Synchronous fluorescence spectra at Δλ = 20 nm of:

(a) TLD (20.0, 40.0, 60.0,100.0, 120.0, 160.0, 200.0) ng mL⁻¹.

(b) TRZ 15.0 ng mL⁻¹.

**Table S1:** Precision data of the proposed method for assessing TLD, DXZ and TRZ in their raw materials.

**Table S2:** ANOVA test for comparing the results across different matrices.

**Table S3:** Matrix effect study for different matrices on TLD, DXZ, and TRZ signals.

**Table S4:** Application of the proposed method for assessing TLD/DXZ and TLD/TRZ in their laboratory-prepared mixtures.

**Table S5:** Application of the proposed method for checking content uniformity testing of TLD (100.0 ng mL^-^¹), DXZ (40.0 ng mL^-^¹), and TRZ (40.0 ng mL^-^¹) in Incont L. A^®^, Dosin^®^, and Itrin^®^ tablets, respectively.

**Table S6:** Application of the proposed method for determining TLD/DXZ and TLD/TRZ in plasma.

**Table S7:** Application of the proposed method for determining TLD, DXZ, and TRZ in tap and river water.

**
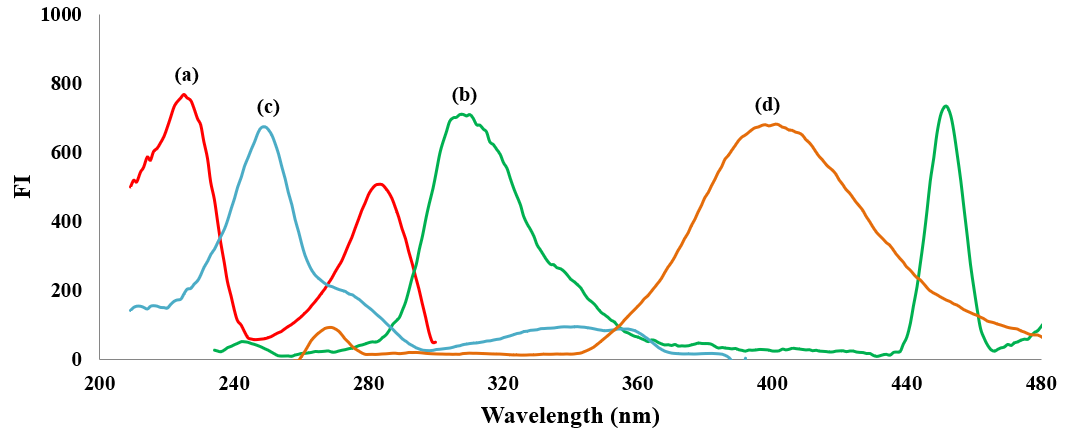

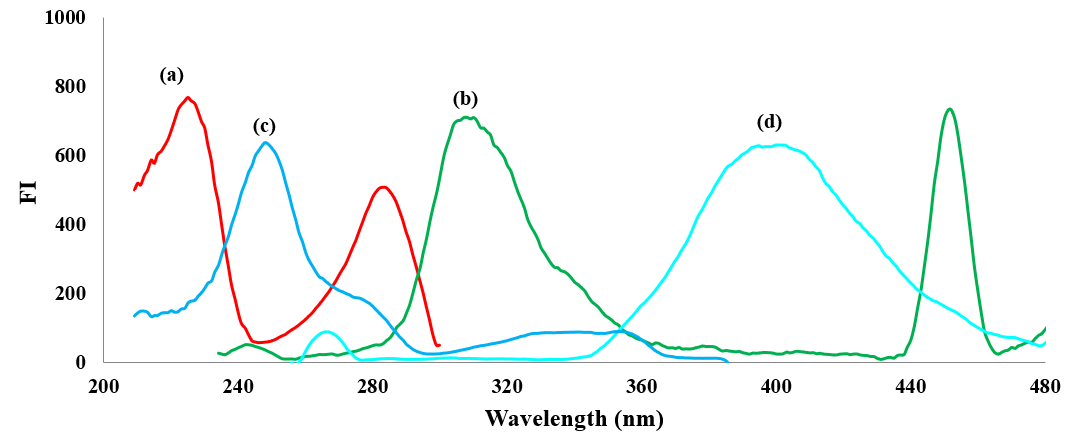
**

**B**
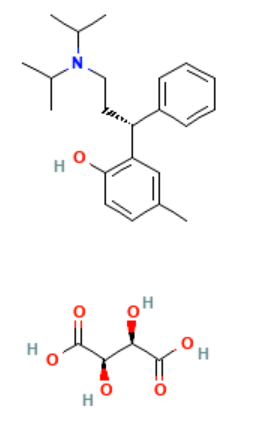


**A**
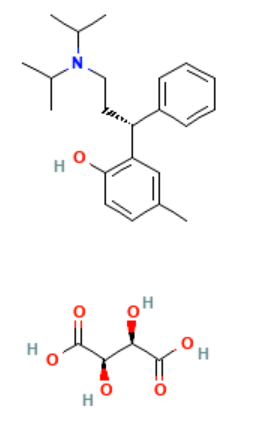


**Fig. S1**

**
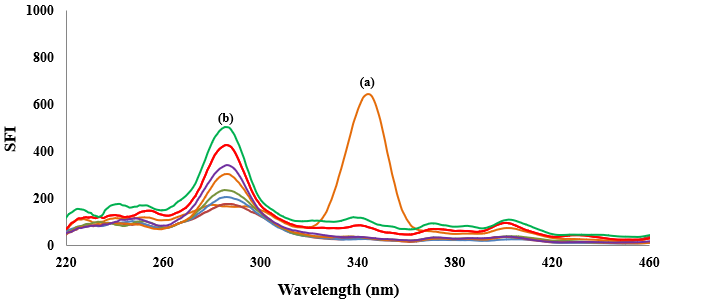

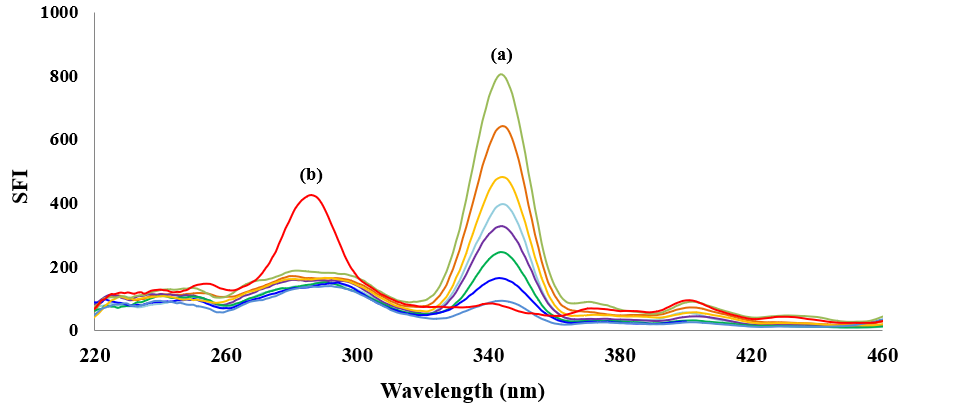
**

**A**
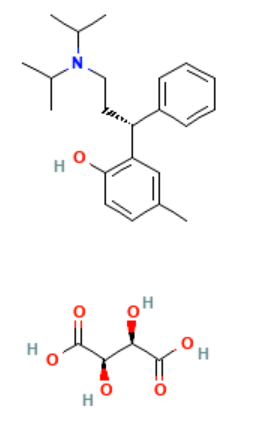


**B**
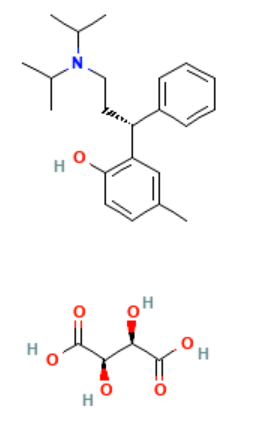


**Fig. S2**

**
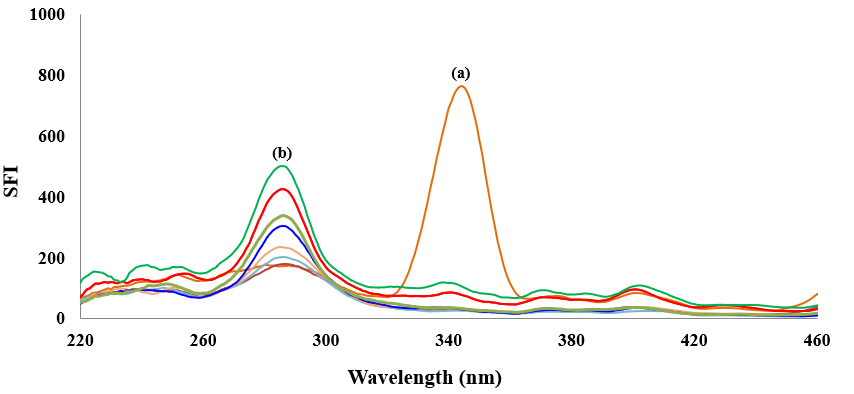

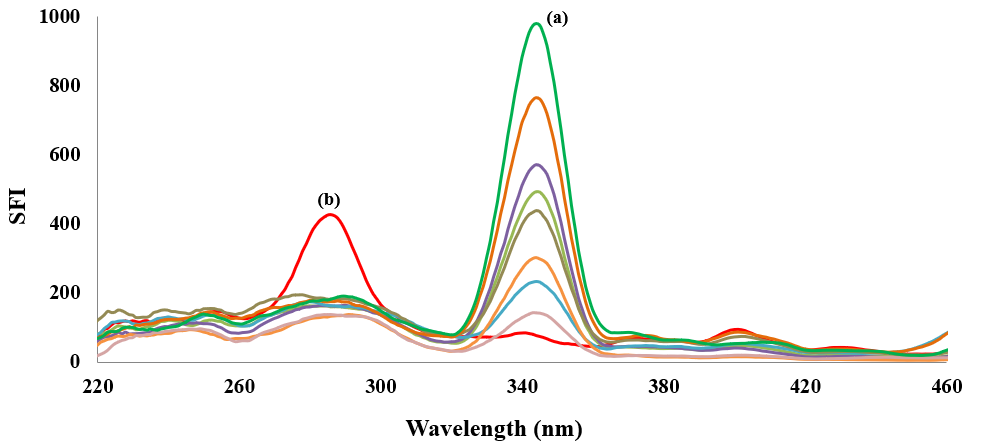
**

**B**
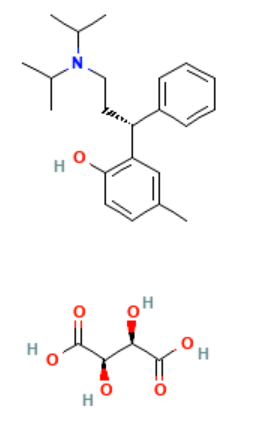


**A**
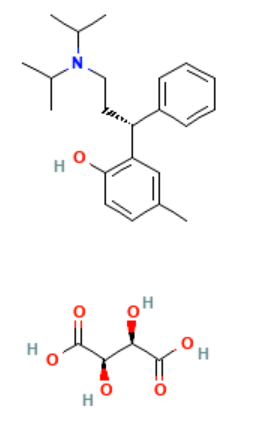


**Fig. S3**

| **TLD** | | | **DXZ** | | | **TRZ** | | |
| --- | --- | --- | --- | --- | --- | --- | --- | --- |
| **Sample concentration** | **Repeatability** | **Intermediate precision** | **Sample concentration** | **Repeatability** | **Intermediate precision** | **Sample concentration** | **Repeatability** | **Intermediate precision** |
| **40.0 ng mL^-1^** | | | **15.0 ng mL^-1^** | | | **15.0 ng mL^-1^** | | |
| **Mean found** $\bar{\boldsymbol{x}}$ | 98.75 | 99.12 | **Mean found** $\bar{\boldsymbol{x}}$ | 100.17 | 100.12 | **Mean found** $\bar{\boldsymbol{x}}$ | 98.01 | 99.29 |
| **± SD** | 1.59 | 1.22 | **± SD** | 0.99 | 1.49 | **± SD** | 1.34 | 0.90 |
| **% RSD** | 1.61 | 1.23 | **% RSD** | 0.99 | 1.49 | **% RSD** | 1.37 | 0.91 |
| **% Error** | 0.92 | 0.70 | **% Error** | 0.57 | 0.86 | **% Error** | 0.77 | 0.52 |
| **100.0 ng mL^-1^** | | | **25.0 ng mL^-1^** | | | **25.0 ng mL^-1^** | | |
| **Mean found** $\bar{\boldsymbol{x}}$ | 101.31 | 101.43 | **Mean found** $\bar{\boldsymbol{x}}$ | 101.96 | 100.51 | **Mean found** $\bar{\boldsymbol{x}}$ | 98.45 | 100.17 |
| **± SD** | 0.84 | 1.27 | **± SD** | 0.22 | 1.69 | **± SD** | 0.40 | 1.34 |
| **% RSD** | 0.83 | 1.25 | **% RSD** | 0.22 | 1.68 | **% RSD** | 0.41 | 1.34 |
| **% Error** | 0.48 | 0.73 | **% Error** | 0.13 | 0.98 | **% Error** | 0.23 | 0.77 |
| **120.0 ng mL^-1^** | | | **40.0 ng mL^-1^** | | | **40.0 ng mL^-1^** | | |
| **Mean found** $\bar{\boldsymbol{x}}$ | 99.58 | 101.45 | **Mean found** $\bar{\boldsymbol{x}}$ | 100.49 | 100.73 | **Mean found** $\bar{\boldsymbol{x}}$ | 99.64 | 99.87 |
| **± SD** | 1.64 | 1.23 | **± SD** | 0.78 | 1.12 | **± SD** | 0.99 | 1.04 |
| **% RSD** | 1.65 | 1.21 | **% RSD** | 0.78 | 1.11 | **% RSD** | 0.99 | 1.04 |
| **% Error** | 0.95 | 0.71 | **% Error** | 0.45 | 0.65 | **% Error** | 0.57 | 0.60 |

**Table S1:**

**Table S2:**

| **Source of Variation** | **SS** | **df** | **MS** | ***F*** | ***P*-value** | ***F* crit** |
| --- | --- | --- | --- | --- | --- | --- |
| **TLD** | | | | | | |
| **Between Groups** | 0.48 | 2 | 0.24 | 0.12 | 0.89 | 4.26 |
| **Within Groups** | 18.66 | 9 | 2.07 |  |  |  |
| **Total** | 19.14 | 11 |  |  |  |  |
| **DXZ** | | | | | | |
| **Between Groups** | 0.14 | 2 | 0.07 | 0.05 | 0.95 | 4.26 |
| **Within Groups** | 11.92 | 9 | 1.32 |  |  |  |
| **Total** | 12.06 | 11 |  |  |  |  |
| **TRZ** | | | | | | |
| **Between Groups** | 0.009 | 2 | 0.004 | 0.003 | 0.99 | 4.26 |
| **Within Groups** | 12.29 | 9 | 1.37 |  |  |  |
| **Total** | 12.29 | 11 |  |  |  |  |

Where SS: The sum of squares quantifies the variability between or within the groups, df: The degrees of freedom, and MS: The mean square

**Table S3:**

| **Analyte** | **Matrix** | **ME%** |
| --- | --- | --- |
| **TLD** | Plasma | - 43.89 |
|  | Tap water | + 1.23 |
|  | River water | + 9.88 |
| **DXZ** | Plasma | - 30.86 |
|  | Tap water | - 3.43 |
|  | River water | - 4.57 |
| **TRZ** | Plasma | - 32.38 |
|  | Tap water | - 1.98 |
|  | River water | + 0.22 |

**Table S4:**

| **Synthetic mixture** | **Amount taken**  **(ng mL^-1^)**  **TLD** | **Amount taken**  **(ng mL^-1^)**  **DXZ** | **%Found**  **TLD** | **%Found**  **DXZ** |
| --- | --- | --- | --- | --- |
| **TLD/DXZ** | 20.00 | 40.00 | 99.62 | 101.13 |
|  | 40.00 | 40.00 | 98.42 | 100.69 |
|  | 60.00 | 30.00 | 99.43 | 102.11 |
|  | 120.00 | 30.00 | 100.80 | 99.73 |
| $\bar{\boldsymbol{x}}$ **± SD** |  | | 99.57 **±** 0.98 | 100.92 **±** 0.99 |
| **Synthetic mixture** | **Amount taken**  **(ng mL^-1^)**  **TLD** | **Amount taken**  **(ng mL^-1^)**  **TRZ** | **%Found**  **TLD** | **%Found**  **TRZ** |
| **TLD/TRZ** | 20.00 | 40.00 | 99.62 | 99.60 |
|  | 40.00 | 40.00 | 100.54 | 100.61 |
|  | 60.00 | 30.00 | 100.84 | 101.16 |
|  | 120.00 | 30.00 | 97.63 | 99.14 |
| $\bar{\boldsymbol{x}}$ **± SD** |  | | 99.66 **±** 1.45 | 100.13 **±** 0.92 |

**Table S5:**

| Parameter | Capsule no. | Percentage of the label claim  TLD | Percentage of the label claim  DXZ | Percentage of the label claim  TRZ |
| --- | --- | --- | --- | --- |
|  | 1 | 98.05 | 101.14 | 104.46 |
|  | 2 | 105.09 | 98.10 | 100.04 |
|  | 3 | 97.83 | 96.06 | 100.04 |
|  | 4 | 100.22 | 96.20 | 100.21 |
|  | 5 | 98.86 | 101.80 | 97.56 |
|  | 6 | 98.59 | 101.14 | 101.10 |
|  | 7 | 99.13 | 100.57 | 99.50 |
|  | 8 | 100.22 | 101.52 | 99.15 |
|  | 9 | 98.05 | 98.29 | 98.97 |
|  | 10 | 97.31 | 101.14 | 103.05 |
| X̅ |  | 99.34 | 99.60 | 100.41 |
| SD |  | 2.24 | 2.23 | 2.02 |
| %RSD |  | 2.25 | 2.24 | 2.01 |
| %Error |  | 0.71 | 0.71 | 0.64 |
| Acceptance value (AV) [38] |  | 5.38 | 5.35 | 4.85 |
| Max. allowed AV (L1) [38] |  | 15.0 | 15.0 | 15.0 |

**Table S6:**

| **Plasma Mixture** | **Amount taken**  **(ng mL^-1^)**  **TLD** | **Amount taken**  **(ng mL^-1^)**  **DXZ** | **%Found**  **TLD** | **%Found**  **DXZ** |
| --- | --- | --- | --- | --- |
| **TLD/DXZ** | 40.00 | 10.00 | 101.45 | 101.14 |
|  | 100.00 | 20.00 | 99.44 | 99.55 |
|  | 120.00 | 30.00 | 96.44 | 99.31 |
|  | 140.00 | 40.00 | 103.00 | 100.43 |
| $\bar{\boldsymbol{x}}$ **± SD** |  | | 100.08 ± 2.83 | 100.11± 0.84 |
| **Plasma Mixture** | **Amount taken**  **(ng mL^-1^)**  **TLD** | **Amount taken**  **(ng mL^-1^)**  **TRZ** | **%Found**  **TLD** | **%Found**  **TRZ** |
| **TLD/TRZ** | 60.00 | 20.00 | 103.39 | 98.60 |
|  | 100.00 | 30.00 | 98.70 | 100.08 |
|  | 120.00 | 40.00 | 99.73 | 102.27 |
|  | 200.00 | 50.00 | 100.18 | 98.74 |
| $\bar{\boldsymbol{x}}$ **± SD** |  | | 100.50 ± 2.02 | 99.92 ± 1.70 |

**Table S7:**

| **Compound** | **Amount taken**  **(ng mL^-1^)** | **Amount found**  **(ng mL^-1^)** | **%Found** |
| --- | --- | --- | --- |
| **TLD**  **Tap water** | 40.00 | 40.235 | 100.59 |
|  | 60.00 | 59.915 | 99.86 |
|  | 100.00 | 99.184 | 99.18 |
|  | 120.00 | 120.646 | 100.54 |
| $\bar{\boldsymbol{x}}$ **± SD** |  | | 100.04 ± 0.66 |
| **DXZ**  **Tap water** | 10.00 | 10.095 | 100.95 |
|  | 20.00 | 19.775 | 98.88 |
|  | 30.00 | 30.143 | 100.48 |
|  | 40.00 | 39.982 | 99.96 |
| $\bar{\boldsymbol{x}}$ **± SD** |  | | 100.07 ± 0.89 |
| **TRZ**  **Tap water** | 10.00 | 9.950 | 99.50 |
|  | 20.00 | 20.223 | 101.12 |
|  | 30.00 | 29.706 | 99.02 |
|  | 40.00 | 40.121 | 100.30 |
| $\bar{\boldsymbol{x}}$ **± SD** |  | | 99.99 ± 0.92 |
| **TLD**  **River water** | 40.00 | 40.604 | 101.51 |
|  | 60.00 | 59.614 | 99.36 |
|  | 100.00 | 98.724 | 98.72 |
|  | 120.00 | 121.055 | 100.88 |
| $\bar{\boldsymbol{x}}$ **± SD** |  | | 100.12 ±1.30 |
| **DXZ**  **River water** | 10.00 | 9.813 | 98.13 |
|  | 20.00 | 20.383 | 101.92 |
|  | 30.00 | 29.830 | 99.43 |
|  | 40.00 | 39.982 | 99.96 |
| $\bar{\boldsymbol{x}}$ **± SD** |  | | 99.86 ± 1.57 |
| **TRZ**  **River water** | 10.00 | 9.918 | 99.18 |
|  | 20.00 | 20.124 | 100.62 |
|  | 30.00 | 30.007 | 100.02 |
|  | 40.00 | 39.954 | 99.89 |
| $\bar{\boldsymbol{x}}$ **± SD** |  | | 99.93 ± 0.59 |
